# Structure-guided design of CPPC-paired disulfide-rich peptide libraries for ligand and drug discovery†

**DOI:** 10.1039/d2sc00924b

**Published:** 2022-05-20

**Authors:** Yapei Wu, Shihui Fan, Meng Dong, Jinjing Li, Chuilian Kong, Jie Zhuang, Xiaoting Meng, Shuaimin Lu, Yibing Zhao, Chuanliu Wu

**Affiliations:** Department of Chemistry, College of Chemistry and Chemical Engineering, The MOE Key Laboratory of Spectrochemical Analysis and Instrumentation, State Key Laboratory of Physical Chemistry of Solid Surfaces, Xiamen University Xiamen 361005 P.R. China chlwu@xmu.edu.cn

## Abstract

Peptides constrained through multiple disulfides (or disulfide-rich peptides, DRPs) have been an emerging frontier for ligand and drug discovery. Such peptides have the potential to combine the binding capability of biologics with the stability and bioavailability of smaller molecules. However, DRPs with stable three-dimensional (3D) structures are usually of natural origin or engineered from natural ones. Here, we report the discovery and identification of CPPC (cysteine–proline–proline–cysteine) motif-directed DRPs with stable 3D structures (*i.e.*, CPPC–DRPs). A range of new CPPC–DRPs were designed or selected from either random or structure–convergent peptide libraries. Thus, for the first time we revealed that the CPPC–DRPs can maintain diverse 3D structures by taking advantage of constraints from unique dimeric CPPC mini-loops, including irregular structures and regular α-helix and β-sheet folds. New CPPC–DRPs that can specifically bind the receptors (CD28) on the cell surface were also successfully discovered and identified using our DRP-discovery platform. Overall, this study provides the basis for accessing an unconventional peptide structure space previously inaccessible by natural DRPs and computational designs, inspiring the development of new peptide ligands and therapeutics.

## Abstract 2

CPPC-paired disulfide-rich peptides with stable 3D structures have been discovered through rational library design and screening, providing unconventional peptide scaffolds for the development of new peptide therapeutics.

## Graphical abstract

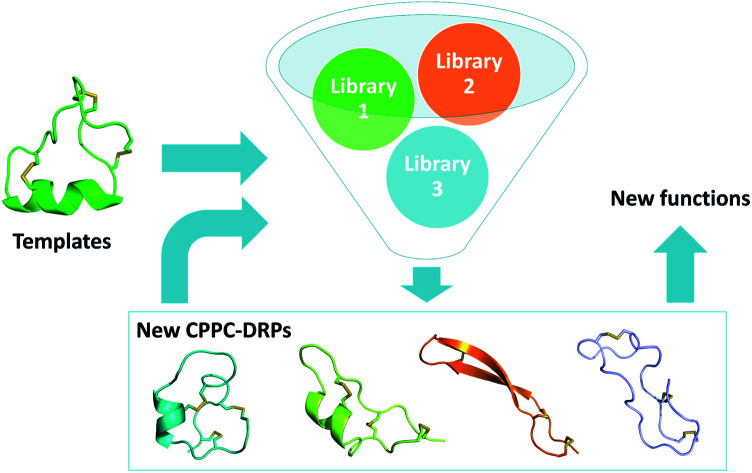

## Introduction

Proteins have finely tuned three-dimensional (3D) structures determining their functions in living organisms. By contrast, short peptides including natural products and those derived from functional motifs of proteins are usually unstructured due to the lack of sufficient structural constraints. Natural disulfide-rich peptides (DRPs) are an exception, as these peptides are constrained through disulfide bonds between cysteine residues.^1,2^ Constraints of disulfide bonds confer unique and stable protein-like 3D structures to these DRPs, which are crucial for their target-binding potency and proteolytic stability.^3–8^ In nature, several unique disulfide-constrained scaffolds, including the cystine-stabilized α/β fold, three-finger toxin fold, and inhibitor cystine knot, have been evolutionally recruited to produce DRPs that can bind a range of targets including cell-surface receptors, ion channels, and proteases.^9–12^ Structures of each scaffold class have been extensively characterized during the last few decades,^13–16^ which provide the structural basis for the engineering of these DRPs for biological and biomedical applications. For instance, new bioactive DRPs can be generated using loop grafting or sequence evolution by taking natural DRPs with known structures as the templates.^17–24^ However, the development of new DRPs is hindered by the limited variety of natural scaffolds. To create DRPs with new structures and functions, computational methods integrated with library screening have been developed.^25–27^ Although powerful, structures of these *de novo* DRPs are limited to neatly arranged α-helix and/or β-sheet folds. Therefore, new methods to create DRPs with diverse 3D structures are still desired.

Recently, we have developed a new class of methods relying on the manipulation of the pattern of thiol-bearing amino acids to *de novo* design new DRPs.^28–32^ Particularly, the orthogonal disulfide-directing effect of CXC and CPPC motifs (C: cysteine; X: any amino acid; P: proline) in peptides enables the design and selection of many new DRPs with diverse disulfide frameworks without recourse to unnatural amino acids.^31,32^ Compared to natural DRPs, these designed disulfide-constrained scaffolds are easier to fold in redox buffers and tolerant to more extensive sequence manipulations, providing a promising and robust platform for peptide ligand and drug discovery.^32^ However, despite the feasibility of developing DRP libraries with all residues in the scaffolds randomized,^32^ the extremely low coverage of random sequences in existing display libraries (*e.g.*, mRNA, ribosomal and phage display) hinders the discovery of new bioactive DRPs due to the limit of library sizes (10^9^ to 10^13^). We argue that revealing the structures of these cysteine-bearing motif-directed DRPs would enable the structure-guided design of display libraries with reasonable coverage of random sequences in a well-defined 3D structure space, which would greatly benefit the development of DRP-based ligands and therapeutics.

In this work, we set out to explore the structure of CPPC motif-directed DRPs (CPPC–DRPs), with the initial aim of revealing structural features compatible to the dimeric CPPC constraint. Solution structures of DRPs selected from phage-display libraries were determined using nuclear magnetic resonance (NMR), revealing the conservativeness and yet variousness of the CPPC–DRPs. Interestingly, the CPPC–DRPs were found to be compatible to not only various irregular structures but also to regular α-helix and β-sheet folds. By exploiting one of the structurally ordered CPPC–DRPs as a template, new structure–convergent libraries were constructed to select peptide ligands towards an extracellular receptor of potential interest to autoimmune diseases and tumor immunotherapy (*i.e.*, CD28). We successfully discovered new CPPC–DRPs that are capable of specifically binding to CD28 on the cell surfaces by strategically combining structure-guided library design and sequential sequence evolution, thus confirming the applicability and robustness of our discovery platform for the development of new peptide therapeutics.

## Results and discussion

### Structural characterization of CPPC–DRPs

**Fig. 1:**
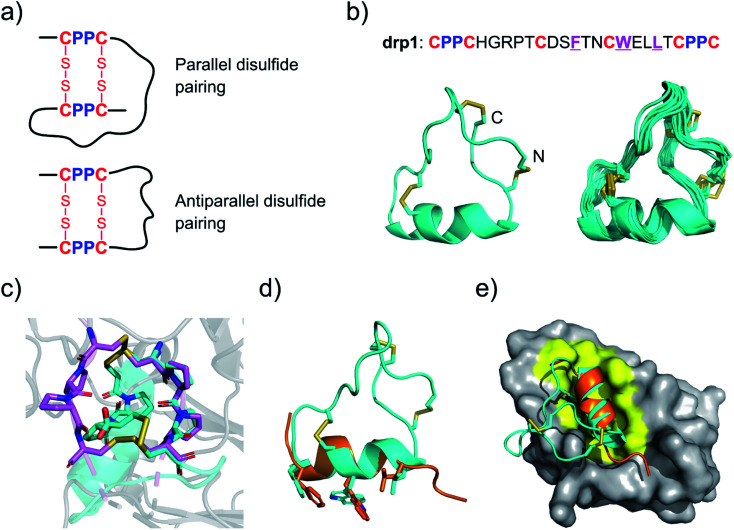
Structures of drp1 and the dimeric CPPC mini-loop. (a) CPPC motif-directed folding of peptides into two different isomers. (b) Solution NMR structures of drp1 (left: cartoon depiction of the lowest-energy structure; right: ensemble of the 15 lowest-energy structures; PDB 7W8K). Cystine disulfides are depicted in sticks. (c) Alignment of the dimeric CPPC mini-loop of drp1 (cyan) with the CPPC motifs in the hinge region of human immunoglobulin G1 (magenta; PDB 5DK3). (d) Alignment of the α-helical region of drp1 with the p53 peptide α-helix (orange). The sidechains of F/W/L are shown in sticks. (e) Complex structure of MDM2 and the p53 peptide α-helix highlighting the binding pocket of MDM2 (PDB 1YCQ).

Two CPPC motifs in peptides tend to form dimeric mini-loops (in parallel or antiparallel orientations, depending on the peptide sequences) after oxidation (Fig. 1a).^32^ This unique phenomenon has been exploited for developing CPPC–DRPs with diverse disulfide frameworks. Previous library screening against the recombinant E3 ubiquitin ligase MDM2 has generated CPPC–DRPs featuring a unique consensus sequence of F–X_3_–W–X_2_–L/I (X denotes any random residues) responsible for target binding.^32^ Thus, we determined the 3D structure of one of the selected CPPC–DRPs (drp1) using ^1^H, ^1^H distance constraints derived from 2D ^1^H, ^1^H NOESY experiments (Fig. 1b; PDB 7W8K). drp1 consists of a stretched loop, a disulfide-constrained turn, and an α-helix locked through an N-/C-terminally dimeric CPPC mini-loop. The dimeric mini-loop has a rigid structure dissimilar to that found in the heave-chain hinge region of human immunoglobulin G1 (Fig. 1c),^33,34^ suggesting the structural adaptability of the CPPC mini-loop in stabilizing the overall 3D structure of the peptide. The α-helical region with the consensus sequence is similar to the previously described p53 peptide α-helix, as evidenced by a root mean square deviation (RMSD) of 0.733 Å between the superimposed backbone atoms (Fig. 1d). This is consistent with expectations, because there is a deep α-helix-binding cleft on the surface of MDM2, which is responsible for specific recognition of α-helical peptides (Fig. 1e).^35^ This is the first structure of this class of peptides. These findings thus indicate that CPPC–DRPs can maintain a stable and unique 3D structure.

### Selection and identification of new CPPC–DRPs

**Fig. 2:**
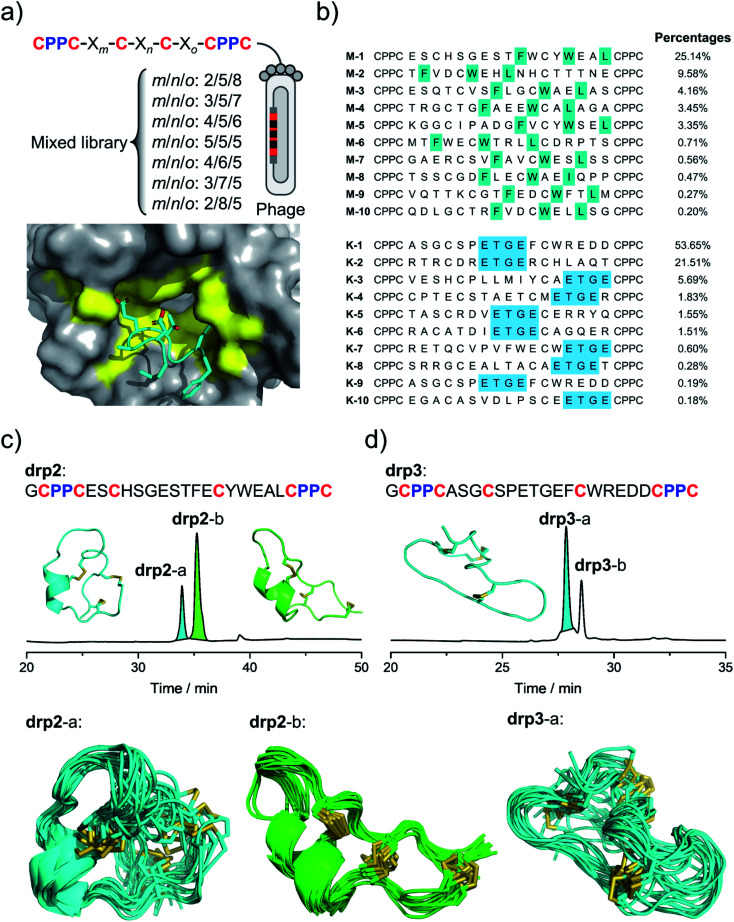
Selection and identification of new CPPC–DRPs. (a) Top: design of a mixed phage-displayed library of CPPC–DRPs (X stands for any amino acid residue, *m*/*n*/*o* denotes the number of amino acids; detailed in Table S1†). Bottom: complex structure of Keap1 and peptides highlighting the binding pocket of Keap1 (PDB 2FLU). (b) Top 10 sequences identified from the selection against MDM2 (top) and Keap1 (bottom). Colors highlight the sequence similarity; percentages indicate the abundance of each sequence. (c) Oxidation and structural characterization of drp2. Top: HPLC chromatogram showing the oxidation of drp2 in a phosphate buffer (pH 7.4, 100 mM) containing 0.5 mM oxidized glutathione (GSSG) and 6 M Gu·HCl. The lowest-energy NMR structure of drp2-a (left, cyan; PDB 7W8O) and drp2-b (right, green; PDB 7W8R). Bottom: the ensembles of the 15 lowest-energy structures for drp2-a (cyan) and drp2-b (green). (d) Oxidation and structural characterization of drp3. Top: HPLC chromatogram showing the oxidation of drp3 in a phosphate buffer (pH 7.4, 100 mM) containing 0.5 mM oxidized glutathione (GSSG) and 6 M Gu·HCl. The lowest-energy NMR structure of drp3-a (cyan; PDB 7W8T). Bottom: the ensemble of the 15 lowest-energy structures of drp3-a (cyan).

After characterizing the structure of drp1, we realized the possibility of selecting CPPC–DRPs with unique 3D structures that can fit into the binding pockets of proteins. Thus, to generate more CPPC–DRPs with a new 3D structure and explore the structure space accessible to CPPC–DRPs, a new phage-displayed library of CPPC–DRPs was constructed by varying the spacing between cysteine residues and panned against MDM2 and another target (the Kelch domain of Keap1)^36^ with a different type of binding pocket (Fig. 2a). After three rounds of selection, the enriched phage pools were sequenced by next-generation sequencing (NGS). In each of the two target selections, we obtained a high degree of sequence enrichment (datasets S1 and S2†). Sequence alignments of the top 50 sequences revealed the consensus sequences (Fig. S1†). MDM2-binding peptides feature a consensus sequence of F–X_3_–W–X_2_–L/I located at different regions of the CPPC–DRP scaffolds (Fig. 2b; top 10 sequences), like that found in drp1. Keap1-binding peptides feature consensus sequences containing an ETGE motif (Fig. 2b and S2†), which is a typical peptide motif mediating the interaction of Nrf2 with Keap1.^37^

The most abundantly enriched sequences from both selections were then synthesized, and their oxidative folding in redox buffers were examined using HPLC. Oxidation of the MDM2-binding peptide (drp2) yields two products (drp2-a and drp2-b) in a ratio of ∼1 : 3 (Fig. 2c). Both products can bind to MDM2 with a subnanomolar affinity (Fig. S3†), as determined using a fluorescence polarization (FP) competition assay described previously.^32^ The structures of the two products were then characterized using NMR (Fig. 2c; PDB 7W8O and 7W8R). Both products can maintain a stable, but different 3D structure with a conserved α-helix responsible for target recognition, corresponding to the parallel and antiparallel dimeric pairing of the two CPPC motifs in the peptide (drp2-a and drp2-b, respectively). Oxidation of the Keap1-bind peptide (drp3) yields two peaks in the HPLC chromatogram in a ratio of ∼3 : 1 (Fig. 2d). Both products can bind to Keap1 with submicromolar affinity (determined using surface plasmon resonance; Fig. S4 and S5†), but the major product exhibits a relatively higher affinity compared to the minor product. Thus, we then determined the 3D structure of the major product using NMR, which exhibits an irregular, loop-extended structure constrained through a parallel CPPC mini-loop (Fig. 2d). These results demonstrated that CPPC–DRPs are chameleonic scaffolds amenable to variations of the 3D structure, and CPPC–DRPs with new structures and functions can be rapidly discovered from the screening of peptide libraries against protein targets featuring different types of binding pockets.

### Structure-based applications of CPPC–DRPs

**Fig. 3:**
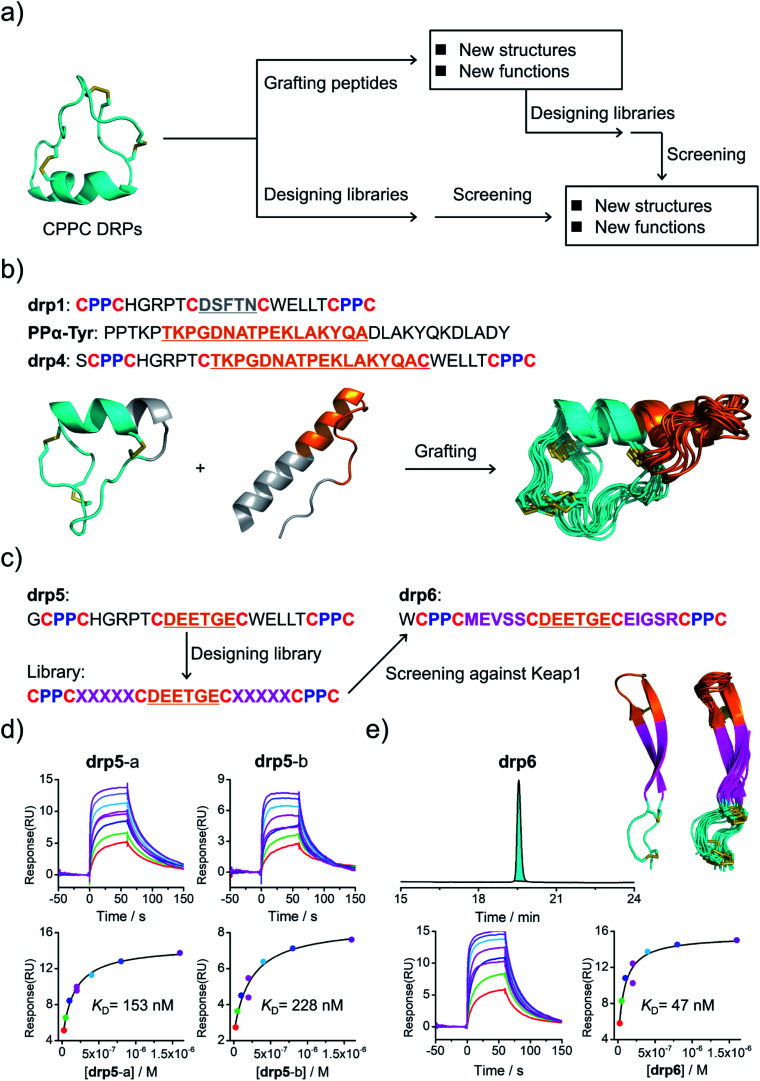
Structure-based applications of CPPC–DRPs. (a) Flow diagram illustrating the applications that can be explored on the basis of structures of CPPC–DRPs, including design of CPPC–DRPs with new structures and functions through epitope grafting and selection of new CPPC–DRPs through library design and screening. (b) Illustration of the design of drp4. From left to right: drp1 as a scaffold shown in cyan; the structural motif of PPα-Tyr (orange) grafted into the drp1 scaffold; structures of the finally designed drp4 (ensemble of the 15 lowest-energy structures; PDB 7W8Z). (c) Schematic view of epitope grafting and design of a secondary library to generate new CPPC–DRPs with Keap1-binding capability. drp5 is generated by grafting an epitope (sequence: DEETGE) into the drp1 scaffold. A secondary library was designed (detailed in Table S1†) by randomizing the residues flanking the grafted epitope. drp6 was the most abundantly enriched sequence from the library screening against Keap1 (dataset S3 and Fig. S9†). (d) SPR sensorgrams showing interaction of Keap1 with the two oxidized products of drp5 (drp5-a and drp5-b) in a concentration-dependent manner (top), and the equilibrium dissociation constant (*K*_D_) values of drp5-a and drp5-b toward Keap1 calculated from SPR measurements (bottom). (e) HPLC chromatogram showing the oxidation of drp6 (50 μM) in a phosphate buffer (pH 7.4, 100 mM) containing 0.5 mM GSSG and 6 M Gu·HCl; the lowest-energy NMR structure of drp6 and the ensemble of the 15 lowest-energy structures; SPR sensorgrams determining the *K*_D_ of drp6 toward Keap1. Note that drp5-a, drp5-b and drp6 used for SPR measurements and structural characterization studies have been purified to a purity of >95% using HPLC (Fig. S10†). Different N-terminal residues were incorporated into the peptides to facilitate synthesis or analysis.

Structural characterization studies have greatly promoted the engineering of natural DRPs for biological and biomedical applications.^17^ The availability of 3D structures of CPPC–DRPs would inspire new explorations on the design and applications of CPPC–DRPs (Fig. 3a). First, it facilitates the use of CPPC–DRPs as templates for designing CPPC–DRPs with new structures and functions through epitope grafting. For example, by taking drp1 as a template, we designed a new peptide by simply replacing the disulfide-constrained turn in drp1 with a loop-α-helix fold in a previously designed miniprotein (PPα-Tyr)^38^ (Fig. 3a and b). The peptide was recombinantly produced in *E. coli* by fusing at the N-terminus of a small ubiquitin-related modifier (SUMO) tag. After cleavage from the SUMO tag, the oxidized peptide (drp4) was purified using HPLC (Fig. S6†). The NMR structure of drp4 reveals that drp4 contains a newly designed and extended α-helix with a minor variation on the arrangement of the dimeric CPPC loop (relative to drp1), and the native conformation of the grafted loop-α-helix fold is largely preserved (Fig. 3b). In addition, the structure of drp4 is extremely stable to thermal treatment, as only a small decline of the circular dichroism (CD) signals reflecting the 3D structure was observed even when the temperature was increased to 95 °C (Fig. S7†). In another example, epitope grafting was explored to generate new CPPC–DRPs with protein-binding capability by replacing the disulfide-constrained turn in drp1 with an epitope (DEETGE)^37^ that can bind Keap1 (Fig. 3a and c). The grafted peptide (drp5; two oxidized products; Fig. S8†) can bind Keap1 with a high-nanomolar affinity (*K*_D_: 153 and 228 nM for the two products respectively; Fig. 3d). Given that CPPC–DRP scaffolds are modifiable, sequence evolution can be explored to increase the target-binding affinity by designing and selecting secondary libraries in which several or even all residues flanking the epitope are mutated into other amino acids. By doing this, new sequences were selected (dataset S3 and Fig. S9†), and the most abundantly enriched sequence was examined. Interestingly, the peptide (drp6) can fold into a quasi-sole oxidized product in redox buffers as shown in the HPLC chromatogram (Fig. 3e; conformed by NMR characterization). The oxidized peptide can bind to Keap1 with a ∼3-fold increased affinity (*K*_D_: 47 nM, Fig. 3e). The NMR structure of drp6 reveals a unique β-sheet fold stabilized through an inter-strand disulfide bond and an antiparallel CPPC mini-loop (Fig. 3e; PDB 7W96). These results suggest the feasibility of combining epitope grafting and library screening for the discovery of new CPPC–DRPs. In addition, we found that new structures derived from precursor scaffolds can be either conserved or subject to broad variations. Particularly, the variability of the dimeric CPPC pairing (parallel or antiparallel) can further amplify the extent of structural variations, enabling access to broad regions of the peptide structure space that were previously inaccessible by natural DRPs and computational designs.

### Construction of structure–convergent peptide libraries

**Fig. 4:**
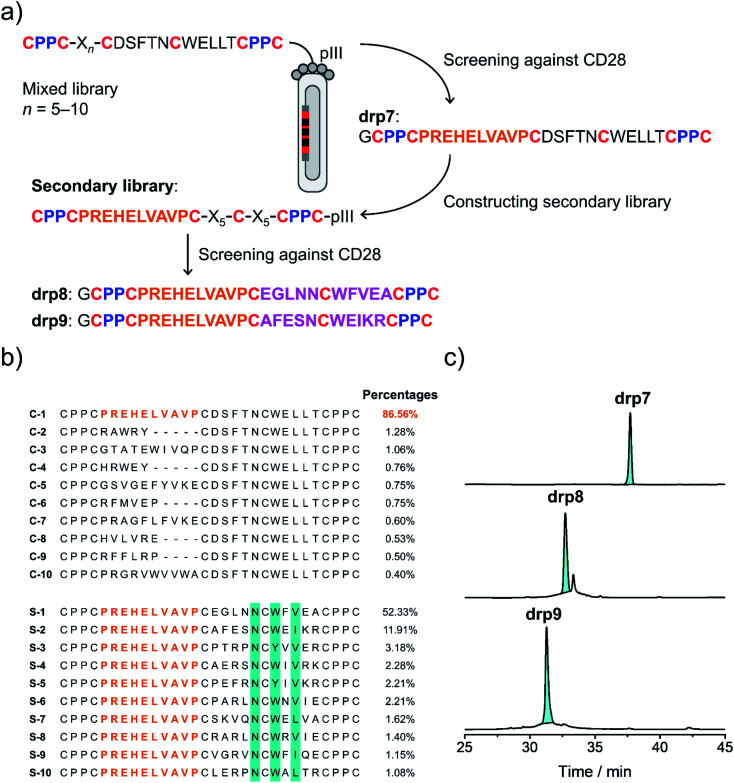
Selection of CPPC–DRPs binding CD28. (a) Schematic view of the library design and screening. The primary library was constructed by encoding one of the loops in drp1 with random sequences of different lengths (detailed in Table S1†). The secondary library was constructed by using the most abundant sequence (drp7) enriched in the primary screening as a template, in which the other two loops were randomly encoded (detailed in Table S1†). drp8 and drp9 are the two most abundant sequences enriched in the secondary library screening. (b) Top 10 sequences selected from the primary (top) and secondary (bottom; conserved residues are highlighted in cyan) library screening, respectively. Percentages indicate the abundance of each sequence. (c) HPLC chromatograms showing the oxidation of drp7, drp8 and drp9 (50 μM) in phosphate buffers (pH 7.4, 100 mM) containing 0.5 mM GSSG and 6 M Gu·HCl.

Secondly, availability of structured CPPC–DRPs enables the construction of structure–convergent peptide libraries (Fig. 3a). Thus, we developed a new phage-displayed peptide library using drp1 as a template, in which the stretched loop at the N-terminus was altered in length and encoded with random amino acids (5–10 residues; Fig. 4a). The successful construction of the library was confirmed by NGS (dataset S4†), indicating the diversity of loop lengths and amino acid composition in the library. In addition, as the library preserves the consensus sequence for binding MDM2, we enriched phages that can bind MDM2 after three rounds of selection and sequenced them by NGS. We observed the enrichment of sequences from all the scaffolds of varied loop lengths (dataset S5†), suggesting the preservation of the α-helical fold in these sequences. Further analysis of the composition of amino acids indicates that most residues in the varied loops are non-conserved (Fig. S11–S16†), albeit with certain exceptions, implying that the CPPC–DRP scaffold featuring the α-helical fold are tolerant to a variety of random sequences in the N-terminal loop. These sequences provide us with a rich source of stable DRP folds worthy of further engineering and development. However, for discovering new ligands to other targets by directly screening the convergent library, it is worth mentioning that the α-helical moiety is not necessary to be preserved. In contrast, a more extended range of variations in structures along with the incorporation of random loops in the library would facilitate new ligand discovery. This structure–convergent peptide library was then applied for discovering new peptide ligands to an extracellular protein target (*i.e.*, CD28).

### Selection of CPPC–DRPs binding CD28

CD28 is a co-stimulatory receptor constitutively expressed on T cells, which is critical for regulating T cell activation and the maintenance of immune homeostasis.^39^ Targeting CD28 has been explored for autoimmune diseases and cancer immunotherapy.^40^ Currently, most explorations are focused on the use of antibodies and antibody fragments for targeting CD28.^41^ However, antibodies and their fragments are large proteins, which might suffer from high production costs, poor thermal stability, and limited tissue penetrability.^42^ In contrast, multicyclic (disulfide-rich) peptides are smaller, more stable in structure, and can be produced with lower costs, representing an underexplored frontier for drug discovery.^43^ However, currently there are no such peptides that have been developed to target CD28, despite the success of discovering or engineering natural DRPs to some other cell surface receptors.^17^ Thus, we then applied the structure–convergent peptide library for selecting CD28 ligands. After four rounds of selection, phages enriched by immobilized CD28 were sequenced by NGS (dataset S6†). Sequence alignments of the top 50 enriched sequences reveal several distinct consensus sequences (Fig. S17;† top 10 sequences are given in Fig. 4b). In contrast, we observed no enrichment of phages for the screening of the aforementioned peptide library with all residues randomized and varied spacing between cysteine residues against CD28, though the library did succeed in selecting peptide ligands for protein targets with well-defined binding pockets. This finding suggests that the structure-convergent peptide library perhaps benefits from the relatively high coverage of random sequences compared to the fully randomized peptide libraries, at least in some instances, to increase the probability of discovering new peptide ligands.

As usual, the most abundantly enriched sequence was synthesized (Fig. 4b), purified using HPLC, and oxidized in GSH/GSSG buffers. Oxidation of the peptide (drp7) yields a quasi-sole product as shown in the HPLC chromatogram (∼100% conversion; Fig. 4c), indicating the superb fold-directing effect of the selected sequence. To assess the contribution of the invariable sequence region in the peptide to CD28 binding and to increase the binding affinity of the peptide, a secondary library with all invariable residues in the primary library replaced by random amino acids was prepared and panned against CD28 (Fig. 4a). We observed remarkable enrichment of phages in the second and third rounds of selection (Fig. S18†), and the enriched phages in the second round of selection were sequenced. Alignments of the top 50 sequences reveal a conserved motif of NCWXV/L/I, with other residues flanking this motif being highly diverse (Fig. 4b and S19†). This conserved motif is coincidently present in the primary library, likely implying that the primary peptide library applied to the panning against CD28 might possess convergent structures favoring the binding of CD28. Then, two of the most abundantly enriched sequences were synthesized (drp8 and drp9), and both peptides can fold efficiently into the expected tricyclic products (later confirmed by NMR structures) in high conversions (∼87% and ∼100%, respectively; Fig. 4c).

### Functional and structural characterization studies of CPPC–DRPs binding CD28

**Fig. 5:**
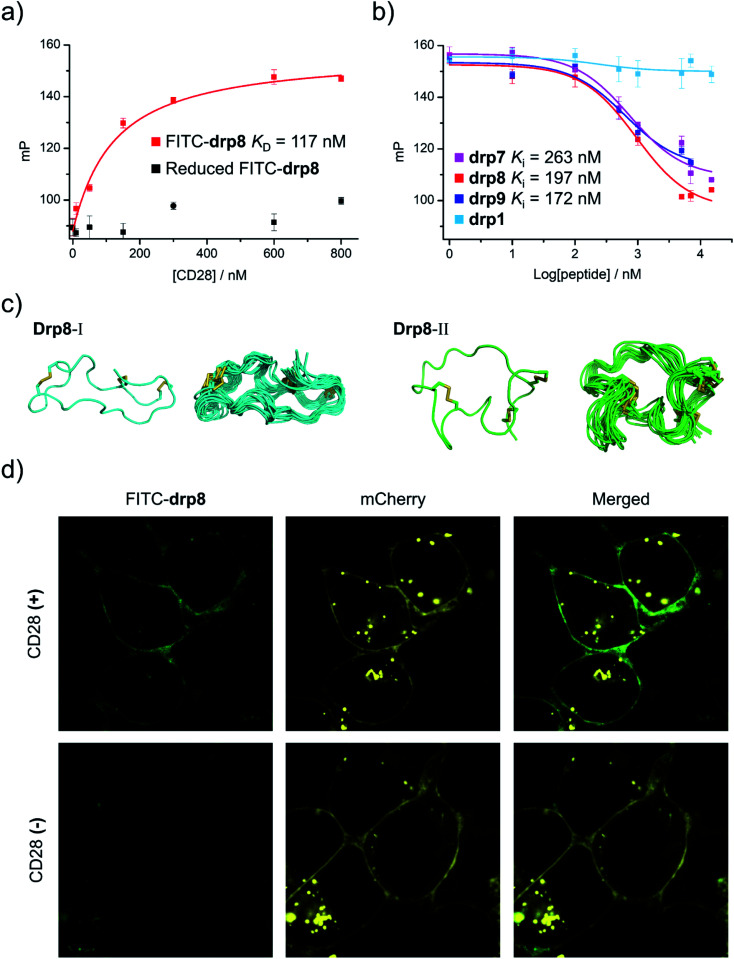
Structural and functional characterization studies of CPPC–DRPs binding CD28. (a) Binding of reduced and oxidized drp8 labeled with fluorescein (FITC) to CD28 determined using a fluorescence polarization (FP) assay. (b) Binding of oxidized drp1, drp7, drp8 and drp9 to CD28 determined by competition with oxidized FITC-drp8 in a FP assay. Note that drp1, drp7, drp8, drp9, and FITC-drp8 used for FP assay and structural characterization studies have been purified to a purity of >95% using HPLC (Fig. S20 and S21†). (c) Solution structures of drp8; the lowest-energy NMR structure of drp8-I and drp8-II and the respective ensemble of the 15 lowest-energy structures. (d) Confocal fluorescence images of FITC-drp8 bound on the cell surfaces. Top: HEK293T cells with recombinant mCherry-CD28 (CD28(+); top) and mCherry (CD28(−); bottom) expressed on the cell surfaces were incubated with 1 μM FITC-drp8 in a medium for 30 min. Green fluorescence was from FITC-drp8; yellow fluorescence was from the mCherry protein.

To assess the binding affinity of these selected peptides to CD28, drp8 was fluorescently labeled by conjugating a fluorescein molecule onto the N-terminus of the peptide. Saturation binding of the fluorescein-labeled drp8 to CD28 was then measured using fluorescence polarization (FP), giving a dissociation constant (*K*_D_) of 117 nM (Fig. 5a), which is over one order of magnitude lower than that between CD28 and its native ligand CD80.^44^ The fluorescence of CD28-bound drp8 is obviously depolarized in the presence of CD80 (Fig. S22†), suggesting the competitive binding of CD80 and drp8 with CD28 and that drp8 is capable of blocking the CD80 and CD28 interaction. The binding of the reduced drp8 with the target is negligible, indicating the important role of the disulfide-constrained 3D structure of the peptide in CD28 binding. The binding affinity of unlabeled peptides (drp7, drp8, drp9, and the negative control drp1) was determined using FP competition assays with fluorescein-labeled drp8 as the probe. It was found that drp7 can also bind with CD28 with nanomolar affinity (Fig. 5b), though it was slightly weaker compared to drp8 (*K*_i_ values: 263 *vs.* 197 nM). drp1 negligibly binds with CD28 (Fig. 5b), indicating that the conserved motif (NCWXL) alone, without the involvement of the selected N-terminal loop, does not possess obvious binding affinity to the target. In addition, drp8 exhibits negligible binding affinity to CTLA4 (Fig. S23†), a CD28 homolog with similar structures and shared ligands,^45^ indicating the CD28-binding specificity of these peptides. These results clearly demonstrated the success of discovering new CPPC–DRPs by exploiting the structure-guided design of a peptide library and the subsequent primary and secondary library screening, which benefit from the sequential evolution of separated peptide fragments in the CPPC–DRP scaffold for ligand discovery.

We further characterized the structure of drp8 using NMR. The cross-peak doublets in both the TOCSY and NOESY spectra show that drp8 adopts two equally populated structural forms in solution (Fig. 5c and S24†). The two structural forms display significantly different chemical shifts for backbone resonance. It is likely that the Cys5–Pro6 peptide bond switches between the two alternative conformations (cis/trans isomerization). The two structures were then determined using ^1^H, ^1^H distance constraints derived from 2D ^1^H, ^1^H NOESY spectra (Fig. 5c; PDB 7WEI and 7WE3, Table S3†). Structural comparison reveals the remarkable difference in the overall 3D structures, though both structures have the same dimeric CPPC disulfide pairing. However, it remains unclear which structure contributes to the binding of drp8 to CD28 before characterizing the structure of the complex.

Finally, we examined the specific binding capability of drp8 to CD28 expressed on the surface of the cells. HEK293T cells transfected with a plasmid expressing the extracellular domain of CD28 fused with an extracellular-targeting signaling sequence, a fluorescent mCherry tag, and a transmembrane domain was constructed for the experiments. Briefly, the transfected cells were incubated with the fluorescein-labeled drp8 for 30 min, followed by washing with culture media to remove the unbound peptide. Confocal fluorescence images were then recorded (Fig. 5d), showing the colocalization of fluorescence from the mCherry tag and the labeled peptide. This result thus clearly demonstrated that the peptide can specifically bind with CD28 on the live cell surface, where a variety of other receptors are present.

## Conclusions

We found that CPPC–DRPs can maintain diverse and stable 3D structures benefiting from the structural constraints of the unique disulfide frameworks. The disclosure of structures of CPPC–DRPs provides opportunities to design and select a variety of CPPC–DRPs with new structures and functions in a more rational manner. We believe that, with the rapid progress of the computational design of constrained peptides, CPPC–DRPs would serve as a new class of multicyclic peptide scaffolds to design new structures and new ligands and therapeutics. New peptide libraries can also be developed by exploiting CPPC–DRPs bearing well-defined 3D structures as templates, which enables the selection of multicyclic peptide ligands and drugs for proteins of therapeutic interest.

Herein, by exploiting a CPPC–DRP with a well-defined structure as a template, we constructed a CPPC–DRP library convergent in structure, which enables us to discover peptide ligands that can bind specifically to CD28 on the surface of live cells. More importantly, the structure-guided design of CPPC–DRP libraries can utilize the sequential sequence evolution strategy to develop peptide ligands with high binding affinity and selectivity. While more and more structures of CPPC–DRPs will be characterized in the near future, based on which new peptide libraries developed, it would greatly benefit the development of *de novo* peptide ligands and therapeutics for a variety of targets. Efforts towards this direction are currently ongoing to extensively explore the potential of CPPC–DRPs as alternative scaffolds to antibodies and traditional cyclic peptides and natural DRPs for drug discovery.

## Data availability

The NMR structures are available in the Protein Data Bank. Other materials may be requested from the corresponding authors.

## Author contributions

Y. W., S. F. and C. W. designed the research. Y. W. and S. F. performed the experiments on peptide synthesis and characterization. Y. W., J. L., S. L. and C. K. performed the experiments on library design and screening. M. D., J. Z. and X. M. performed the experiments on protein expression and fluorescence imaging. S. F. characterized the peptide structures. Y. W., S. F. and C. W. analyzed the data. Y. W., S. F., Y. Z. and C. W. wrote and revised the manuscript. C. W. supervised the research. All authors reviewed and approved the manuscript.

## Conflicts of interest

The authors declare no competing interests.

## Supplementary Material

SC-013-D2SC00924B-s001

SC-013-D2SC00924B-s002

SC-013-D2SC00924B-s003

SC-013-D2SC00924B-s004

SC-013-D2SC00924B-s005

SC-013-D2SC00924B-s006

SC-013-D2SC00924B-s007

SC-013-D2SC00924B-s008

SC-013-D2SC00924B-s009
