# Supplementary material for: Structure-guided design of CPPC-paired disulfide-rich peptide libraries for ligand and drug discovery
Source: Chem Sci. 2022 May 20;13(26):7780–9. doi: 10.1039/d2sc00924b (PMC9258321; doi:10.1039/d2sc00924b)
Supplement: SC-013-D2SC00924B-s009 [file SC-013-D2SC00924B-s009.pdf]

Structure-Guided Design of CPPC-Paired Disulfide-Rich Peptide Libraries for Ligand and Drug Discovery

Yapei Wu,[†] Shihui Fan,[†] Meng Dong, Jinjing Li, Chuilian Kong, Jie Zhuang, Xiaoting Meng, Shuaimin Lu, Yibing Zhao, and Chuanliu Wu*

Department of Chemistry, College of Chemistry and Chemical Engineering, The MOE Key Laboratory of Spectrochemical Analysis and Instrumentation, State Key Laboratory of Physical Chemistry of Solid Surfaces, Xiamen University, Xiamen, 361005, P.R. China.

*Corresponding Author; Email: chlwu@xmu.edu.cn

List of Supplementary Material

Experimental section

Table S1. Information of phage libraries

Table S2. Information of peptides

Figure S1. Top 50 peptide sequences in consensus with the Library-1 targeting MDM2

Figure S2. Top 50 peptide sequences in consensus with the Library-1 targeting Keap1

Figure S3. Characterization and affinity experiments of **drp2**

Figure S4-S5. Characterization and affinity experiments of **drp3**

Figure S6-S7. Chromatograms, CD spectra and NMR of **drp4**

Figure S8. Chromatograms of oxidation of **drp5**

Figure S9-S10. Top 50 peptide sequences in consensus with the Library-2 targeting Keap1

Figure S11-S16. Peptide sequences in consensus with the Library-3 targeting MDM2

Figure S17. Top 50 peptide sequences in consensus with the Library-3 targeting CD28

Figure S18. Screening of secondary library (Library-4) against CD28

Figure S19. Top 50 peptide sequences in consensus with the Library-4 targeting CD28

Figure S20-S21. Characterization of **drp7**, **drp8**, **drp9** and FITC-**drp8**

Figure S22. Binding of CD80 to CD28

Figure S23. Binding of **drp8** to CTLA-4

Table S3. Experimental NMR data

Figure S24. Two-dimensional ^{1}H-^{1}H TOCSY and ^{1}H-^{1}H NOESY spectra of **drp8**

Experimental section

Materials and instruments

Primers for constructing the libraries were purchased from Genscript (Nanjing, China). Klenow fragment, *Sfi I*, *Not I*, T4 DNA Ligase, 20 bp DNA Ladder and DL5000 DNA Marker were bought from Takara (Beijing, China). Recombinant protein CD28 (Cat: 11524-HCCH) and CD80 (Cat: 10698-H08H) was supplied by Sino Biological (Beijing, China). Ampicillin (Amp), agar, ammonium persulfate and 4S Red Plus were bought from Sangon Biotech (Shanghai, China). EZ-Link™ Sulfo-NHS-LC-Biotin, Dynabeads™ M-280 Streptavidin, Dynabeads™ M-280 Tosyl-activated and neutravidin were purchased from Thermo Fisher Scientific (Shanghai, China). Fmoc-protected amino acids and MBHA resins used in peptide synthesis were purchased from GL Biochem (Shanghai, China). Acetonitrile (ACN), trifluoroacetic acid (TFA), glutathione reduced (GSH), glutathione oxidized (GSSG), dithiothreitol (DTT), tris (2-carboxyethyl) phosphine hydrochloride (TCEP) were supplied by Sigma-Aldrich (Beijing, China). Acetonitrile-D3 was purchased from Cambridge Isotope Laboratories (Beijing, China). Deuterium oxide (D_2O) was purchased from Energy Chemical (Shanghai, China). TG1 *E. coli* strain was purchased from Beyotime (Shanghai, China). All the chemicals used to prepare the PB (18.6 mM $NaH_2PO_4·2H_2O$, 81.4 mM $Na_2HPO_4·12H_2O$, pH 7.4), 1×PBS, 1×TBE (890 mM Tris-HCl, 20 mM $Na_2EDTA·2H_2O$, 890 mM boric acid,), 3 M NaAc (pH 5.2), 100 mM NaAc (pH 4.5), 1×TE (10 mM Tris-HCl, 1 mM EDTA disodium salt dihydrate, pH 8.0), 1 mM EDTA (EDTA disodium salt dihydrate, pH 8.0), sodium dodecyl sulfate (SDS), binding buffer (10 mM Tris-HCl, 150 mM NaCl, 10 mM $MgCl_2$, 1 mM $CaCl_2$, pH 7.4), washing buffer (binding buffer containing 0.1 % Tween-20), blocking buffer (binding buffer containing 0.3 % Tween-20 and 3 % (w/v) BSA), elution buffer (50 mM glycine, pH 2.2), neutralization buffer (1 M Tris-HCl, pH 8.0), PEG/NaCl (20 % PEG-8000 (w/v), 2.5 M NaCl) and 2-YT medium (16 g tryptone, 10 g yeast extract, 5 g NaCl per 1 L H_2O) were purchased from Sigma-Aldrich (Beijing, China), Sangon Biotech (Shanghai, China) or Sinopharm Chemical Reagent (Beijing, China).

Recombinant plasmids used for library construction were transformed into TG1 *E. coli* using electroporation (Bio-Rad). Peptides were synthesized by solid phase peptide synthesis (SPPS) using CEM Liberty Blue™ automated microwave peptide synthesizer and analyzed by high performance liquid chromatography (HPLC) (SHIMADZU system). Bruker autoflex max MALDI-TOF mass spectrometry was used for identifying peptides. Peptides were quantified using HITACHI U-3900H UV/Vis spectrometer. The concentrations of proteins or nucleic acid were determined by Nanodrop (Thermo Fisher Scientific). Fluorescence anisotropy was measured in a 96 well flat-bottom OptiPlate black plate by Infinite® 200 PRO

multimode microplate readers (TECAN). NMR experiments were recorded at 298 K on Bruker AVANCE III 850 MHz equipped with a cryogenic triple-resonance probe.

Library construction

All peptide libraries were constructed as described previously[1]. Oligonucleotides (chapter 2.1) coding peptides were co-incubated with the reverse primer (5'−CACCGGCGCACCTTGCGGCCGC−3') in extended system containing Klenow fragment. The two double-stranded products of DNA and phagemid vector (pCantab 5E) were then digested with *Sfi I* (8 h, 50°C) and *Not I* (8 h, 37°C). For each library, the purified *Sfi I/Not I*-digested DNA fragments were ligated into the phagemid and electroporated into the E. *coli* TG1 cells. After that, the library capacity was evaluated by counting the clone number of gradient diluted plates (Table S2 shows exogenous peptides that were displayed on the surface of phages).

Biotinylation of protein

Protein was biotinylated at a concentration of 1 μM with a 5-fold molar excess of Sulfo-NHS-LC-biotin in PBS, pH 7.4 at room temperature for 30 min. The biotinylated protein was purified on a HiTraq™ 5 mL using PBS, pH 7.4.

Phage screening

Phages encoded exogenous peptides were recovered from 2-YT medium supernatant with PEG/NaCl precipitation and PBS re-suspension. The phage titer was then measured, and the phages were blocked in blocking buffer for 30 min. The streptavidin magnetic beads (or neutravidin-coated magnetic beads) binding with biotinylated protein were also blocked using the blocking buffer for 120 min. The magnetic beads were incubated with the phage peptide library at room temperature for 30 min. After washing nine times with washing buffer and twice with binding buffer, the phages were eluted from magnetic beads with glycine-hydrochloric acid solution (50 mM glycine, pH 2.2). The phage amplification and titer measurement were conducted to make a preparation for the next round of panning. Phage titer was determined after each round of panning to monitor the results of screening. After three or four rounds of panning, the enriched phages were sequenced using next-generation sequencing.

Sequence identification by next-generation sequencing (NGS)

Phage vectors extracted from *E. coli* TG1 were stored as glycerol stocks. The DNA was isolated with a commercial plasmid purification kit (TIANGene® Tianjin, China). Phage vector DNA was amplified by PCR. The PCR reaction contained final concentrations of 250 μM dNTPs, 100 nM primer, 100 ng phage vector as temple, 1 unit Taq polymerase and 10 μL of 10×Taq buffer. The mixture solution was filled up to 100 μL with water. The following program was used: initial denaturation for 60 s at 95°C, 12 cycles of 30 s at 95°C, 30 s at 65°C, 30 s at 72°C, and final elongation for 5 min at 72°C. The product was purified

from 2% agarose gel (containing 0.005% v/v of 4S Red Plus Nucleic Acid Stain) used a commercial agarose gel purification kit (E.Z.N.A® Gel Extraction Kit, OMEGA BIO-TEK, USA). Then, Novogene Co., Ltd. was commissioned for sequence identification. The data were processed and analyzed using MatLab scripts[2, 3].

Synthesis of peptides

All peptides were synthesized on a CEM Liberty Blue automated microwave peptide synthesizer by standard Fmoc solid-phase chemistry on a Rink Amide MBHA resin (0.025 mmol scale). Coupling reactions were performed with a Fmoc-AA-OH (5 eq., 0.2 M solution in DMF), DIC (5 eq., 0.25 M solution in DMF) and Oxyma Pure (5 eq., 1.0 M solution in DMF). Fmoc groups were removed using a 20% (v/v) solution of piperidine in DMF. Following the synthesis, peptidyl resins were cleaved by treating with a mixture of TFA/EDT/thioanisole/Phenol/H_2O (87.5/2.5/5/2.5/2.5 v/v, 5 mL) for 4 h at 37°C. The resin was removed by filtration and the peptides were precipitated with cold diethyl ether (30 mL), collected by centrifugation (6000 rpm at 4°C, 4 min). The precipitated peptides were resuspended and washed three times with diethyl ether (30 mL each time). Then, the crude peptides were purified by preparative HPLC. The peptides isolated from preparative HPLC can be purified again to ensure the purity (>95%).

Oxidative folding of peptides

In a typical experiment, reduced peptide (50 μM) was dissolved in 100 mM phosphate buffer (500 μL, pH 7.4) containing 6 M Gu·HCl, 0.5 mM GSSG and 10% DMSO (v/v). The reaction mixture was stirred for 12 h at 37°C. After the reaction is complete, the oxidized peptide was further injected directly in HPLC using a gradient of 15–80% acetonitrile (0.1% TFA) and water (0.1% TFA) over 60 min with a C-18 column. After analyzed by MALDI-TOF MS, the oxidized peptide was purified and lyophilized to yield a white powder.

Circular dichroism (CD) spectra characterization

Circular dichroism (CD) wavelength and temperature scans were recorded for thermal denaturation experiments. The **drp4** was prepared at 61 μM final concentration in pure water. Wavelength scans from 190 nm to 260 nm were recorded at 25°C, 45°C, 75°C and 95°C, and temperature were carried out at a heating rate of 5°C/min in 1 mm path length cuvettes.

Fluorescence polarization (FP) assay

(a) Fluorescence polarization binding assay. The binding of oxidized FITC-**drp8** to CD28 was measured by adding dilutions of CD28 to a fixed concentration of the fluorescent peptide in PBS (pH 7.4). The solutions were added to wells of a 96-well plate to reach a total assay volume of 125 μL. The final concentrations of oxidized FITC-**drp8** were 25 nM and the CD28 ranged from 10 nM to 800 nM. After 10

min incubation at room temperature, the fluorescence anisotropies were measured using a plate reader (E_{ex}= 485 nm, E_{em}=535 nm). All of the measurements were recorded in triplicate. In the fluorescence polarization assays measuring the binding of reduced FITC-**drp8** to CD28 and the binding of oxidized FITC-**drp8** to CTLA-4, the fluorescence anisotropies were recorded in the same way as described above.

Dissociation constants (K_D) were determined by nonlinear regression analysis of anisotropies versus the concentrations of the protein using the following equation:

$$\boldsymbol{Y = A_1 + (A_2 - A_1) \times \left\{\frac{(X+C+K_D)-\sqrt{(X+C+K_D)^2-4\times X\times C}}{2\times C}\right\}} \quad \textbf{(1)}$$

X: the concentration of protein, ***Y***: the measured fluorescence anisotropy, ***C***: the concentration of fluorescent peptide, $\boldsymbol{A_1}$: the bottom plateaus of anisotropy, $\boldsymbol{A_2}$: the anisotropy signal in the presence of saturating concentrations of CD28, $\boldsymbol{K_D}$**:** the dissociation constant.

(b) Competition fluorescence polarization assay. The binding of **drp8** to CD28 was measured by adding dilutions of **drp8** to premixed oxidized FITC-**drp8** and CD28 in PBS (pH 7.4). The solutions were added to wells of a 96-well plate to reach a total assay volume of 125 μL. The final concentrations of **drp8** ranged from 1 nM to 1.5 μM, and the final concentrations of oxidized FITC-**drp8** and CD28 were 25 nM and 225 nM, respectively. After 10 min incubation at room temperature, the plate was read using a plate reader (E_{ex}= 485 nm, E_{em} = 535 nm). In the competition fluorescence polarization assay measuring the binding of **drp1, drp7** and **drp9** to CD28, the fluorescence anisotropies were recorded in the same way as described above. The binding of peptides to MDM2 was evaluated using a method reported previously[1].

The polarization data were fitted with the equation (2) in Origin, which is based on one-site competitive model. The inhibition constant K_i was calculated by equation (3)[4].

$$\boldsymbol{Y = A_2 + \frac{A_1 - A_2}{1+10^{\left(X-logEC_{50}\right)}}} \quad \textbf{(2)}$$

$$\boldsymbol{K_i = [EC]_{50}/\left(\frac{[L]_{50}}{K_D} + \frac{[P]_0}{K_D} + 1\right)} \quad \textbf{(3)}$$

X**:** the log molar concentration of the oxidized peptides, ***Y***: the measured fluorescence anisotropy, $\boldsymbol{A_1}$**:** the top plateaus of anisotropy, $\boldsymbol{A_2}$**:** the bottom plateaus of anisotropy, $\boldsymbol{[EC]_{50}}$: the concentration of the oxidized peptide at 50% inhibition, $\boldsymbol{[L]_{50}}$**:** the concentration of the FITC-labeled peptide at 50% inhibition, $\boldsymbol{[P]_0}$**:** the concentration of the free protein at 0% inhibition, $\boldsymbol{K_D}$**:** the dissociation constant of fluorescent peptide to protein.

(c) The binding of CD80 to CD28 was measured by adding dilutions of CD80 to premixed oxidized FITC-**drp8** and CD28 in PBS (pH 7.4). The solutions were added to wells of a 96-well plate to reach a total assay volume of 100 μL. The final concentrations of CD80 ranged from 50 nM to 500 nM, and the final concentrations of oxidized FITC-**drp8** and CD28 were 25 nM and 100 nM, respectively. After 20 min incubation at room temperature, the plate was read using a plate reader (E_{ex}= 485 nm, E_{em} = 535 nm).

Surface plasmon resonance (SPR) assay

The affinity of peptides to Keap1 was measured using Biacore T200 (GE healthcare) at 25°C. At first, Keap1 was linked to a Biacore CAP chip via the interaction of biotin and avidin (Biotinylation of Keap1 occurs through the preceding reaction, Biacore CAP chip was performed through a Biotin CAPture Kit (GE healthcare)). Then, different concentrations of peptides were used in experiment (25, 50, 100, 200, 400, 800, 1600 nM) at a flow rate of 30 μL/min. The binding affinity and kinetics constants were quantified by a multi-cycle affinity method.

NMR experiments

NMR samples were dissolved in 80% perdeuterated acetonitrile and 20% H_2O for **drp1**, 75% perdeuterated acetonitrile and 35% H_2O for **drp2-a**, 60% perdeuterated acetonitrile and 40% H_2O for **drp8**, 50% perdeuterated acetonitrile and 50% H_2O for **drp3** and **drp6**, 40% perdeuterated acetonitrile and 60% H_2O for **drp2-b** and **drp4** (The solution conditions depend on the dispersion of cross-peaks). All of NMR samples were prepared in a final concentration of about 1 mM. NMR experiments were carried out at 298 K on Bruker AVANCE III 850 MHz equipped with a cryogenic triple-resonance probe. Two dimensional (2D) ^{1}H, $^{15}N/^{13}C$ HSQC spectra were recorded to obtain chemical shifts of heavy atoms in backbone and side-chains. 2D ^{1}H-^{1}H TOCSY (mixing time, 80 ms) and 2D ^{1}H-^{1}H COSY spectra were acquired to the sequential assignment of peptides, and 2D ^{1}H-^{1}H NOESY spectra with a mixing time of 300 ms were carried out for structure calculation. The NMR data were processed using NMRPipe/NMRDraw and analyzed using NMRFAM-SPARKY[5]. More than 95% of all NOE cross-peaks were assigned manually. The backbone dihedral angle restraints were obtained using programs TALOS-N based on chemical shifts of backbone resonances[6]. The distance constraints were deduced from volume integration of NOE cross-peaks and carried out with ARIA2.3.2 and CNS1.21[7, 8]. All of the ensemble of 15 lowest-energy structures were generated from a total of 150 structures in last iteration for each run. The structures were visualized in PyMol, and the quality of structures was analyzed using PROCHECK[9].

Laser confocal cell imaging

(a) Plasmid construction. Two plasmids (Plasmid-A: pCAG-CD28-mCherry-PDGFR; Plasmid-B: pCAG-mCherry-PDGFR) were purchased from Tsingke for cell labeling experiments. Plasmid-A encoded an exogenous N-terminal the murine Igκ-chain leader sequence followed by the extracellular domain of CD28 (Asn19-Gln159) and mCherry with the platelet derived growth factor receptor (PDGFR) transmembrane domain. Plasmid-B encoded exogenous mCherry with the platelet derived growth factor receptor (PDGFR) transmembrane domain.

(b) Labeling CD28 on mammalian cells. HEK293T cells were maintained in DMEM medium (high glucose) supplemented with 10% FBS at 37℃ in a humidified atmosphere containing 5% CO2. HEK293T cells were seeded in a culture dish with glass slide diameter of 20 mm at 1.5×104 cells/dish. After around 24 h, HEK293T cells were transiently transfected with pCAG-CD28-mCherry-PDGFR using PEI. After 24 h, 1 μM of FITC-drp8 in DMEM was added to cells and incubated for 30 min. Cells were then washed four times with 1 mL DMEM. The experiment on pCAG-mCherry-PDGFR was carried out in the same way as a control. Confocal fluorescent images were then recorded in a Leica TCS SP8 confocal microscope system.

Information of phage libraries

DNA sequences of the oligonucleotides (5' to 3') used for vector generation and library cloning. N represents any of the 4 nucleotides and K represents thymidine (T) and guanosine (G). Four phage libraries were constructed by different oligonucleotides which encode different exogenous peptides. Table S1 shows the information of displayed peptides and capacity of different libraries.

Oligonucleotides of Library-1:

5'−TCGCGGCCCAGCCGGCCATGGCATGCCCGCCGTGCNNKNNKTGCNNKNNKNNKNNKNNKTGCNNKNNKNNKNNKNNKNNKNNKNNKTGCCCGCCGTGCGCGGCCGCAAGGTGCGCCGGTG−3'

5'−TCGCGGCCCAGCCGGCCATGGCATGCCCGCCGTGCNNKNNKNNKTGCNNKNNKNNKNNKNNKTGCNNKNNKNNKNNKNNKNNKNNKTGCCCGCCGTGCGCGGCCGCAAGGTGCGCCGGTG−3'

5'−TCGCGGCCCAGCCGGCCATGGCATGCCCGCCGTGCNNKNNKNNKNNKTGCNNKNNKNNKNNKNNKTGCNNKNNKNNKNNKNNKNNKTGCCCGCCGTGCGCGGCCGCAAGGTGCGCCGGTG−3'

5'−TCGCGGCCCAGCCGGCCATGGCATGCCCGCCGTGCNNKNNKNNKNNKNNKTGCNNKNNKNNKNNKNNKTGCNNKNNKNNKNNKNNKTGCCCGCCGTGCGCGGCCGCAAGGTGCGCCGGTG−3'

5'−TCGCGGCCCAGCCGGCCATGGCATGCCCGCCGTGCNNKNNKNNKNNKTGCNNKNNKNNKNNKNNKNNKTGCNNKNNKNNKNNKNNKTGCCCGCCGTGCGCGGCCGCAAGGTGCGCCGGTG−3'

5'−TCGCGGCCCAGCCGGCCATGGCATGCCCGCCGTGCNNKNNKNNKTGCNNKNNKNNKNNKNNKNNKNNKTGCNNKNNKNNKNNKNNKTGCCCGCCGTGCGCGGCCGCAAGGTGCGCCGGTG−3'

5'−TCGCGGCCCAGCCGGCCATGGCATGCCCGCCGTGCNNKNNKTGCNNKNNKNNKNNKNNKNNKNNKNNKTGCNNKNNKNNKNNKNNKTGCCCGCCGTGCGCGGCCGCAAGGTGCGCCGGTG−3'

Oligonucleotides of Library-2:

5'−TCGCGGCCCAGCCGGCCATGGCATGCCCGCCGTGCNNKNNKNNKNNKNNKTGCGATGAAGAAACTGGTGAATGCNNKNNKNNKNNKNNKTGCCCGCCGTGCGCGGCCGCAAGGTGCGCCGGTG−3'

Oligonucleotides of Library-3:

5'−TCGCGGCCCAGCCGGCCATGGCATGTCCTCCGTGTNNKNNKNNKNNKNNKTGTGACTCTTTCACCAACTGCTGGGAACTGCTGACCTGCCCGCCGTGTGCGGCCGCAAGGTGCGCCGGTG−3'

5'−TCGCGGCCCAGCCGGCCATGGCATGTCCTCCGTGTNNKNNKNNKNNKNNKNNKTGTGACTCTTTCACCAACTGCTGGGAACTGCTGACCTGCCCGCCGTGTGCGGCCGCAAGGTGCGCCGGTG−3'

5'−TCGCGGCCCAGCCGGCCATGGCATGTCCTCCGTGTNNKNNKNNKNNKNNKNNKNNKTGTGACTCTTTCACCAACTGCTGGGAACTGCTGACCTGCCCGCCGTGTGCGGCCGCAAGGTGCGCCGGTG−3'

5'−TCGCGGCCCAGCCGGCCATGGCATGTCCTCCGTGTNNKNNKNNKNNKNNKNNKNNKNNKTGTGACTCTTTCACCAACTGCTGGGAACTGCTGACCTGCCCGCCGTGTGCGGCCGCAAGGTGCGCCGGTG−3'

5'−TCGCGGCCCAGCCGGCCATGGCATGTCCTCCGTGTNNKNNKNNKNNKNNKNNKNNKNNKNNKTGTGACTCTTTCACCAACTGCTGGGAACTGCTGACCTGCCCGCCGTGTGCGGCCGCAAGGTGCGCCGGTG−3'

5'−TCGCGGCCCAGCCGGCCATGGCATGTCCTCCGTGTNNKNNKNNKNNKNNKNNKNNKNNKNNKNNKTGTGACTCTTTCACCAACTGCTGGGAACTGCTGACCTGCCCGCCGTGTGCGGCCGCAAGGTGCGCCGGTG−3'

Oligonucleotides of Library-4:

5'−TCGCGGCCCAGCCGGCCATGGCATGCCCGCCATGCCCGCGCGAACACGAACTGGTTGCAGTTCCGTGTNNKNNKNNKNNKNNKTGTNNKNNKNNKNNKNNKTGCCCGCCGTGCGCGGCCGCAAGGTGCGCCGGTG−3'

Table S1 Displayed peptides and capacity of different libraries.

Phage library	Displayed peptides	Capacity
Library-1	CPPCXXCXXXXXCXXXXXXXXCPPC<br>CPPCXXCXXXXXXXXCXXXXXCPPC<br>CPPCXXXCXXXXXCXXXXXXXCPPC<br>CPPCXXXCXXXXXXXCXXXXXCPPC<br>CPPCXXXXCXXXXXCXXXXXXCPPC<br>CPPCXXXXCXXXXXXCXXXXXCPPC<br>CPPCXXXXXCXXXXXCXXXXXCPPC	1.1×10^9
Library-2	CPPCXXXXXCDEETGECXXXXXCPPC	2.8×10^8
Library-3	CPPCXXXXXCDSFTNCWELLTCPPC<br>CPPCXXXXXXCDSFTNCWELLTCPPC<br>CPPCXXXXXXXCDSFTNCWELLTCPPC<br>CPPCXXXXXXXXCDSFTNCWELLTCPPC<br>CPPCXXXXXXXXXCDSFTNCWELLTCPPC<br>CPPCXXXXXXXXXXCDSFTNCWELLTCPPC	3.1×10^8
Library-4	CPPCPREHELVAVPCXXXXXCXXXXXCPPC	6.6×10^8

Information of peptides

Table S2 Sequence and molecular weight of peptides.

Number	Sequence	Molecular weight Reduced	Molecular weight Oxidized
**drp1**	H-CPPCHGRPTCDSFTNCWELLTCPPC-NH_2	2780.1043	2774.0934
**drp2**	H-GCPPCESCHSGESTFWCYWEALCPPC-NH_2	2892.2830	2886.2364
**drp3**	H-GCPPCASGCSPETGEFCWREDDCPPC-NH_2	2746.0470	2740.9363
**drp4**	H-SCPPCHGRPTCTKPGDNATPEKLAKYQACWELLTCPPC-OH	4117.8773	4111.8303
**drp5**	H-GCPPCHGRPTCDEETGECWELLTCPPC-NH_2	2933.3440	2928.1047
**drp6**	H-WCPPCMEVSSCDEETGECEIGSRCPPC-NH_2	2947.3280	2941.0404
**drp7**	H-GCPPCPREHELVAVPCDSFTNCWELLTCPPC-NH_2	3418.4741	3412.4270
**drp8**	H-GCPPCPREHELVAVPCEGLNNCWFVEACPPC-NH_2	3371.4482	3365.4012
**drp9**	H-GCPPCPREHELVAVPCAFESNCWEIKRCPPC-NH_2	3472.5465	3466.4966
**FITC-drp8**	FITC-(βA)GCPPCPREHELVAVPCEGLNNCWFVEACPPC-NH_2	3812.5267	3806.4798

Top 50 peptide sequences in consensus with the Library-1 targeting MDM2

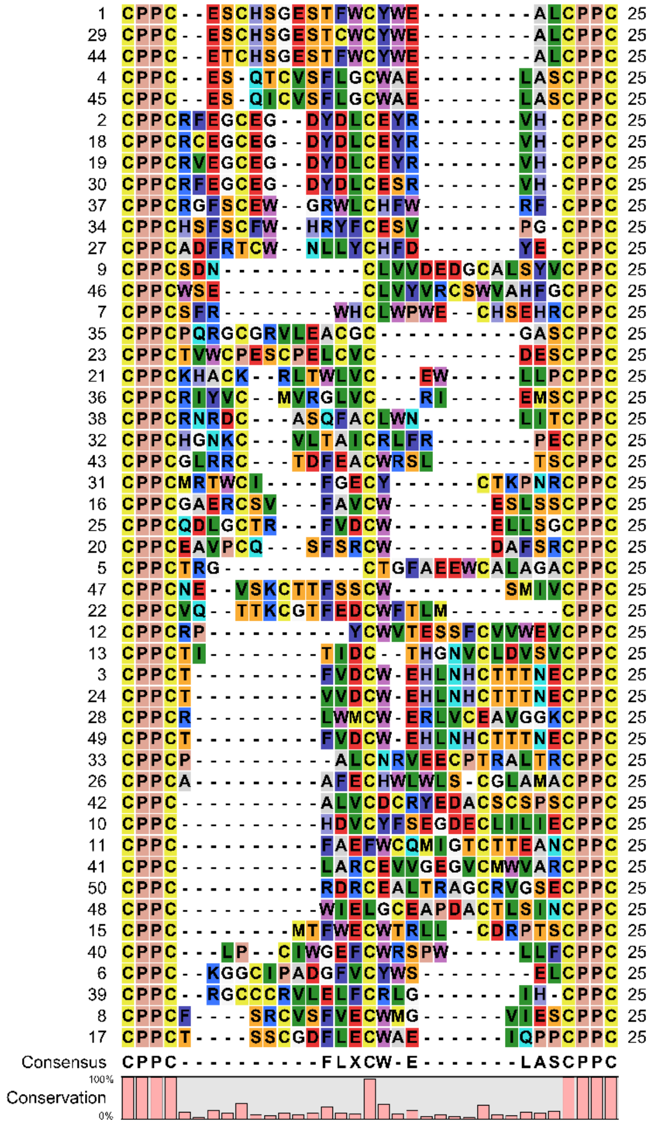

Figure S1. Selected top 50 peptide sequences in consensus with the Library-1 targeting MDM2. Alignments of sequences reveal a conserved motif of F-X_3-W-X_2-L/I.

Top 50 peptide sequences in consensus with the Library-1 targeting Keap1

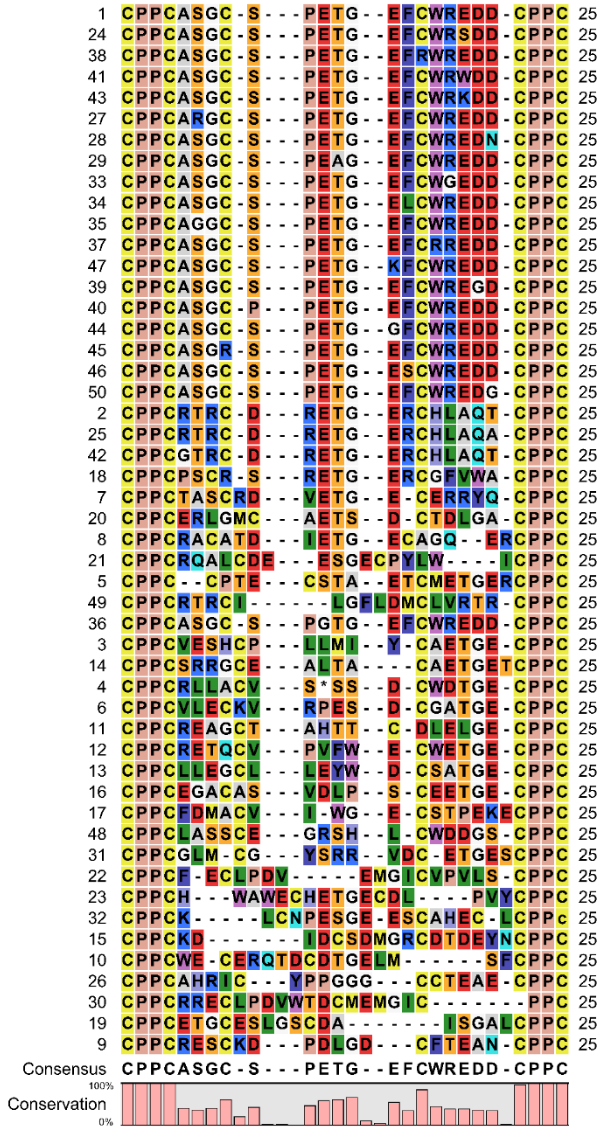

Figure S2. Selected top 50 peptide sequences in consensus with the Library-1 targeting Keap1. Alignments of sequences reveal a conserved motif of ETGE.

Characterization and affinity experiments of drp2

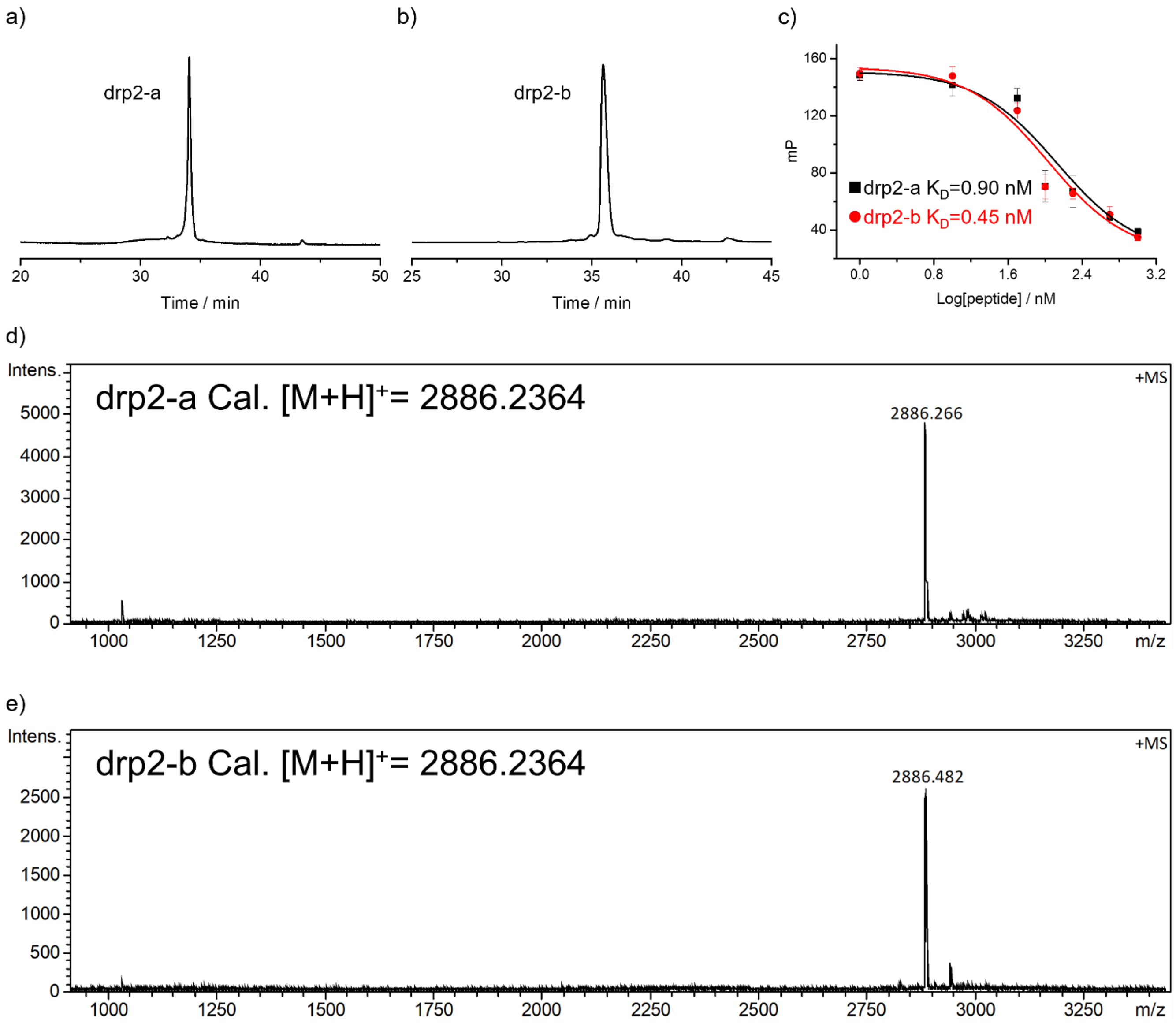

Figure S3. a) The oxidation product **drp2**-a used for the FP assays and NMR was purified using HPLC to a purity of >95%. b) The oxidation product **drp2**-b used for the FP assays and NMR was purified using HPLC to a purity of >95%. c) FP competition curves showing the binding of **drp2**-a and **drp2**-b to MDM2. d) Mass spectrum of **drp2**-a. e) Mass spectrum of **drp2**-b.

Characterization and affinity experiments of drp3

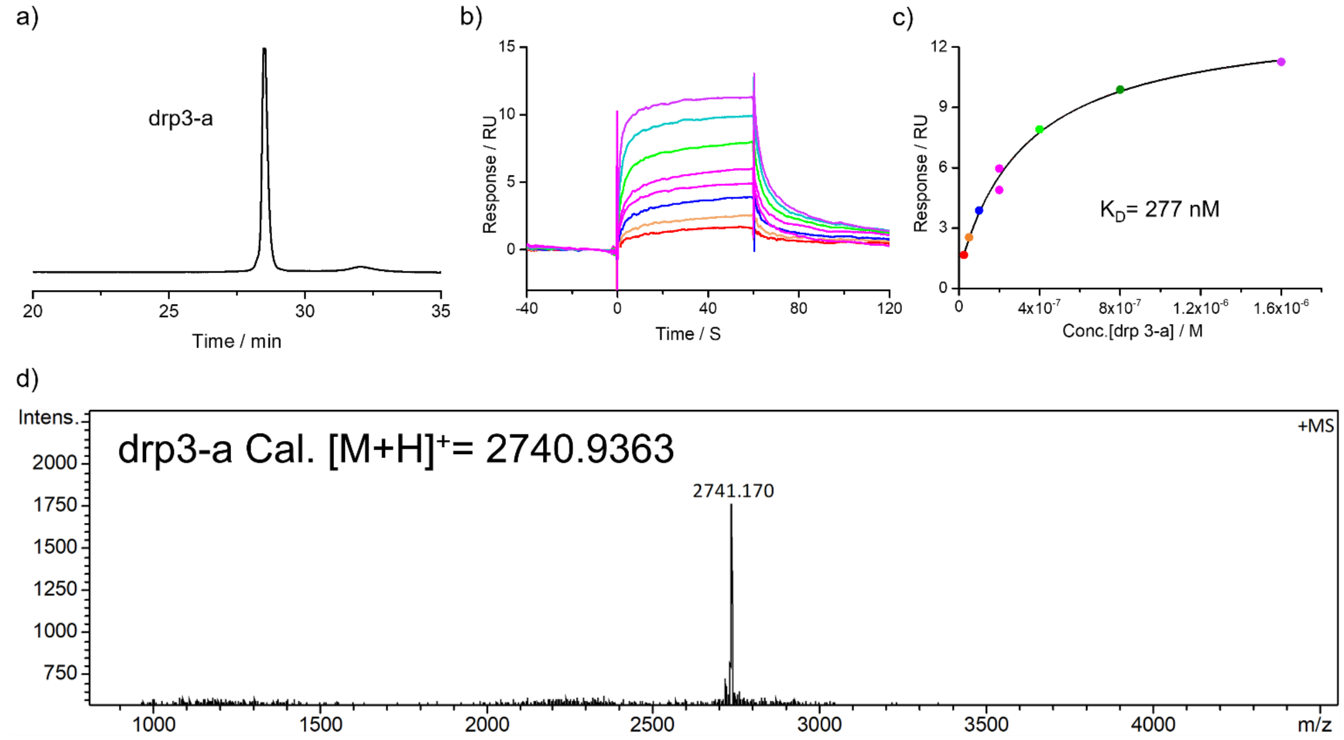

Figure S4. a) The oxidation product **drp3**-a used for SPR sensorgrams and NMR was purified using HPLC to a purity of >95%. b) SPR sensorgrams showing the interaction of Keap1 with the **drp3**-a in a concentration-dependent manner c) The equilibrium dissociation constant (K_D) value of **drp3**-a toward Keap1 calculated from SPR measurements. d) Mass spectrum of **drp3**-a.

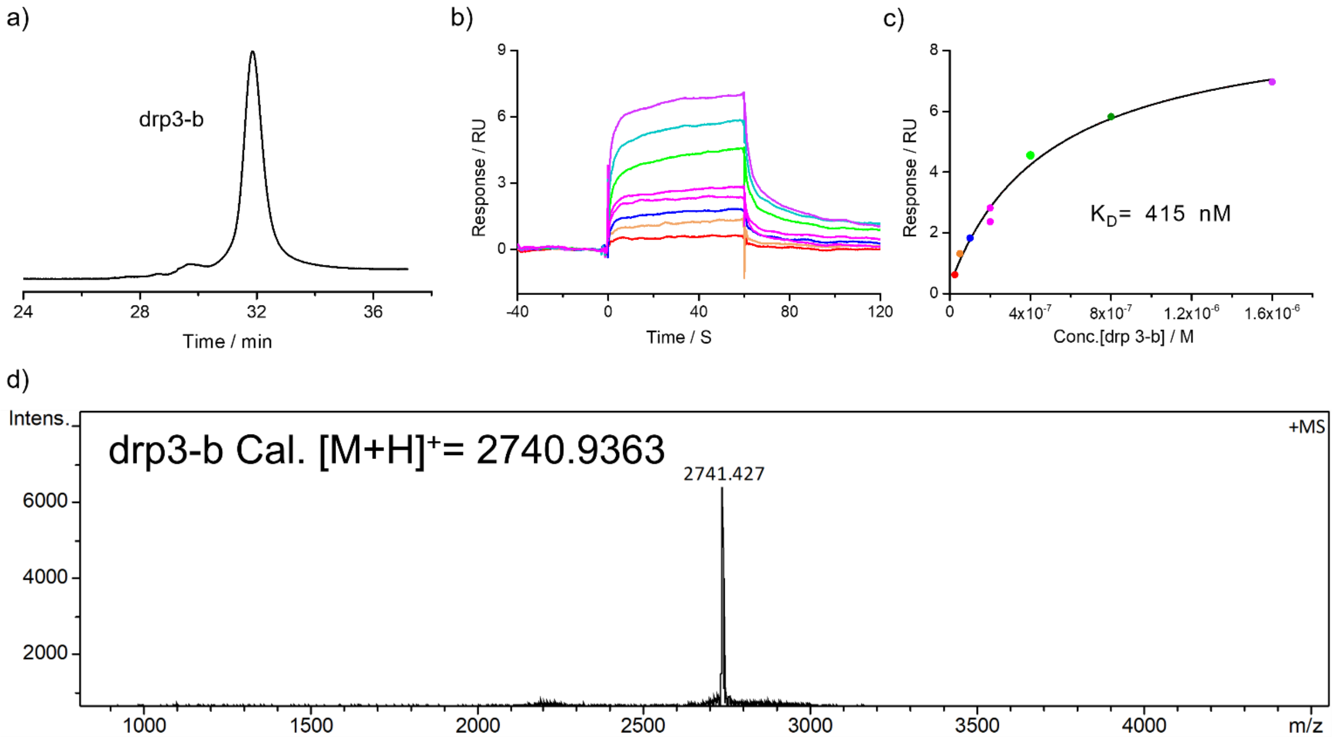

Figure S5. a) The oxidation product **drp3**-b used for SPR sensorgrams was purified using HPLC to a purity of >95%. b) SPR sensorgrams showing the interaction of Keap1 with the **drp3**-b in a concentration-dependent manner c) The equilibrium dissociation constant (K_D) value of **drp3**-b toward Keap1 calculated from SPR measurements. d) Mass spectrum of **drp3**-b.

Chromatograms, CD spectra and NMR of drp4

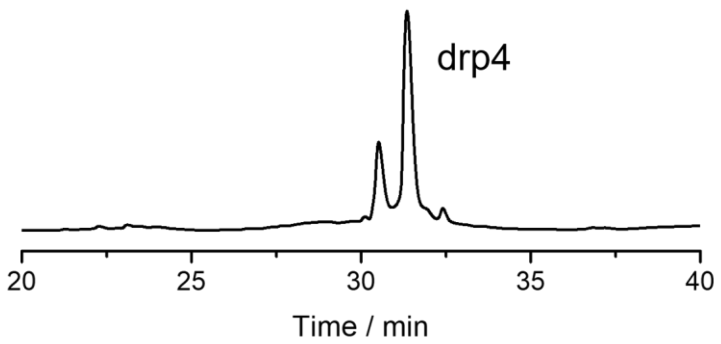

Figure S6. HPLC chromatogram showing the oxidation of **drp4**. The major peak of oxidized **drp4** was purified and further analyzed by circular dichroism (CD) and NMR.

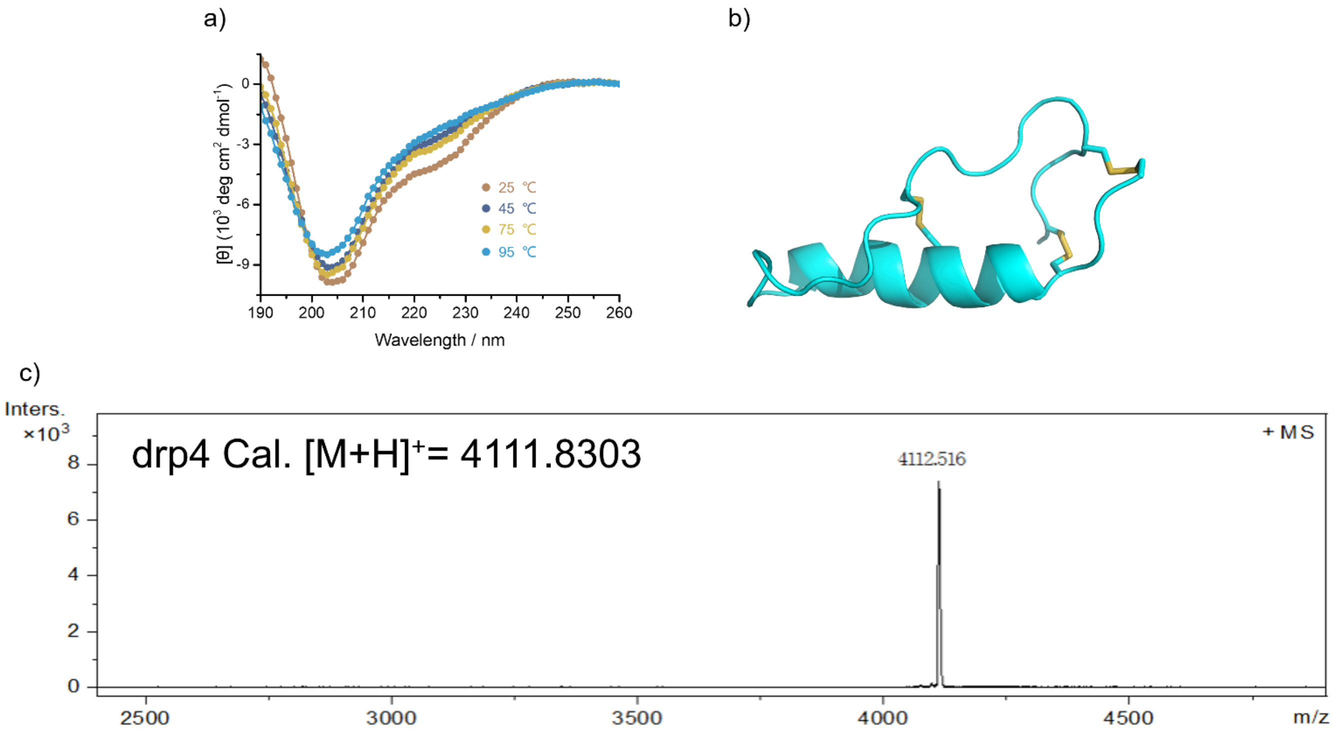

Figure S7. a) CD spectra of **drp4** (50 µM) in water. b) Cartoon depiction of the lowest-energy structure of **drp4**. c) mass spectrum of **drp4**.

Chromatograms of oxidation of drp5

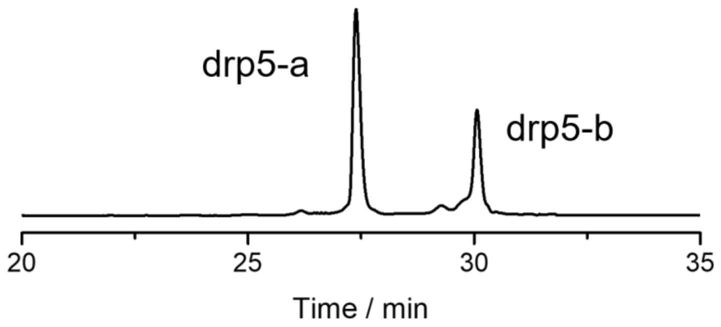

Figure S8. Chromatogram showing the oxidation of **drp5**. HPLC chromatogram showing the oxidation of **drp5** in a phosphate buffer (pH 7.4, 100 mM) containing 0.5 mM oxidized glutathione (GSSG) and 6 M Gu·HCl.

Top 50 peptide sequences in consensus with the Library-2 targeting Keap1

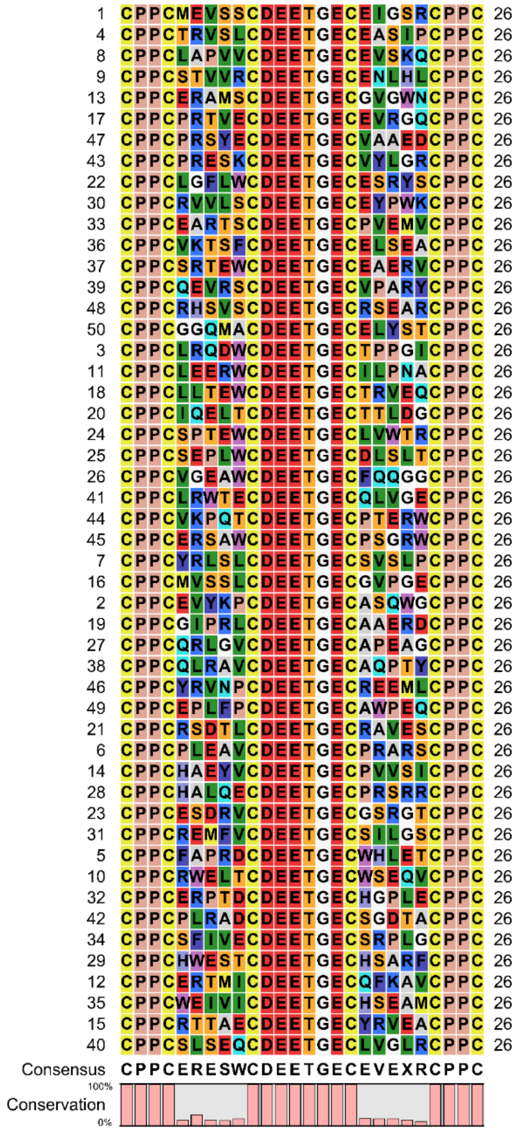

Figure S9. Selected top 50 peptide sequences in consensus with the Library-2 targeting Keap1.

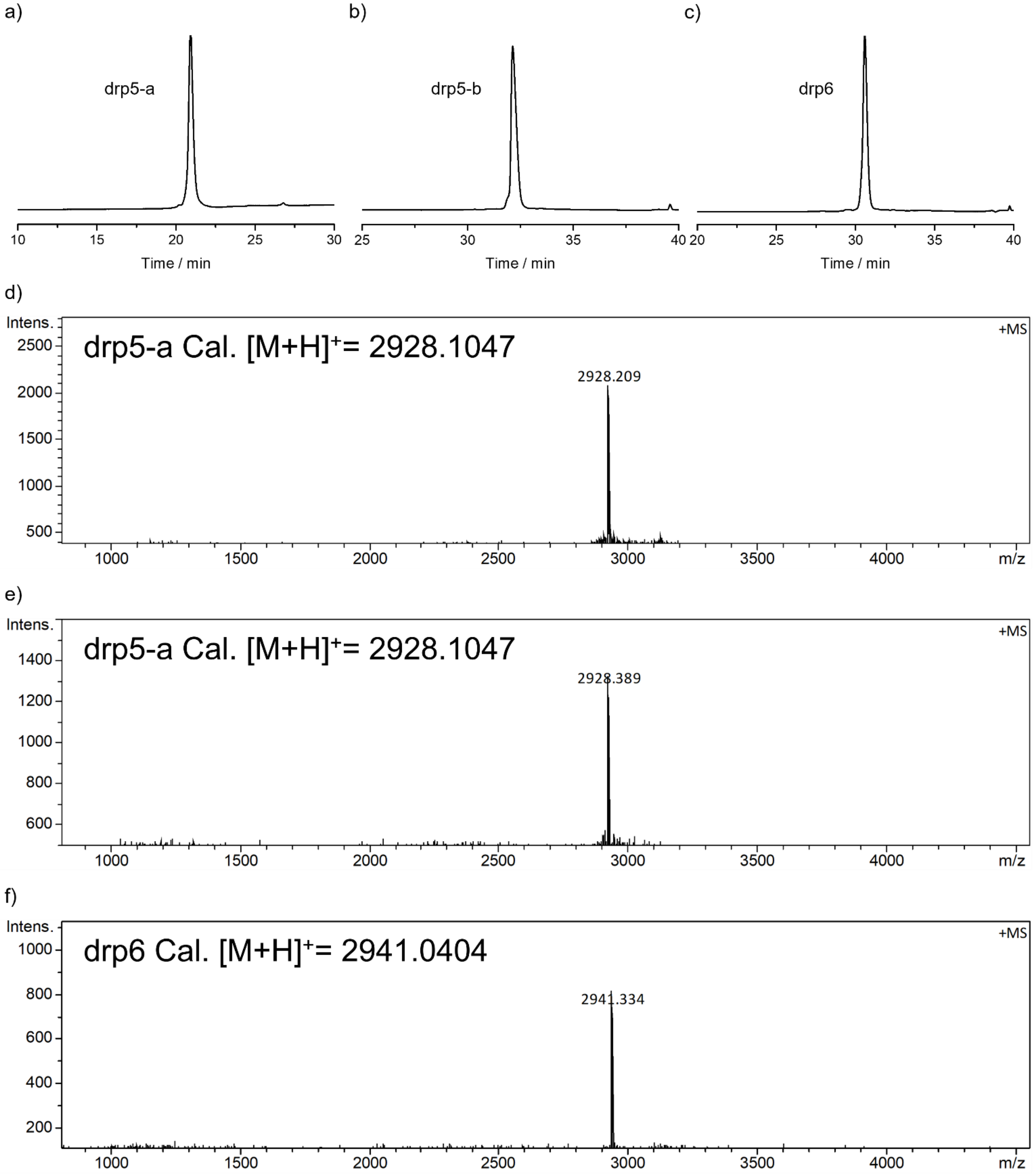

Figure S10. a) The oxidation product **drp5**-a used for SPR sensorgrams was purified using HPLC to a purity of >95%. b) The oxidation product **drp5**-b used for SPR was purified using HPLC to a purity of >95%. c) The oxidation product **drp6**-a used for SPR sensorgrams and NMR was purified using HPLC to a purity of >95%. d) Mass spectrum of **drp5**-a. e) Mass spectrum of **drp5**-b. f) Mass spectrum of **drp6**.

Peptide sequences in consensus with the Library-3 targeting MDM2

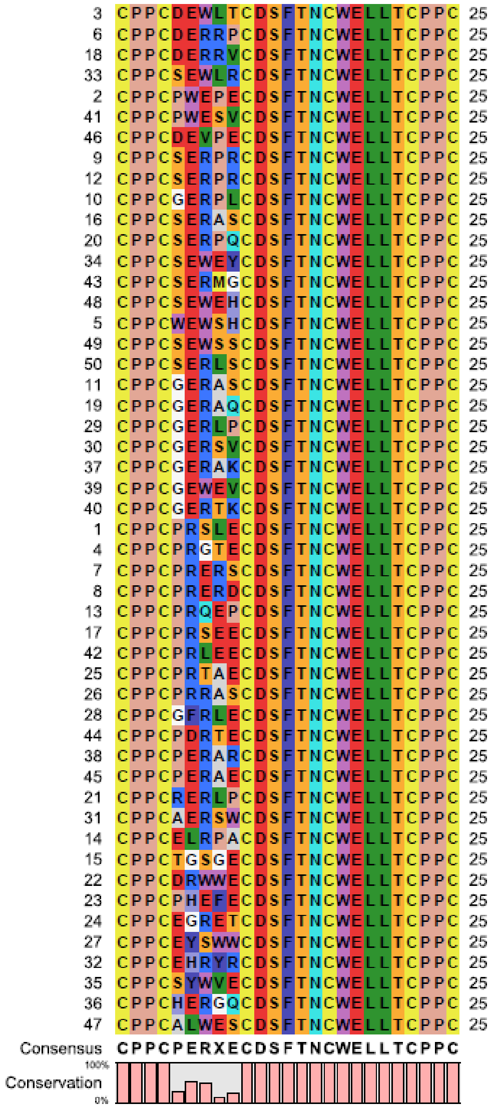

Figure S11. Selected top 50 peptide sequences (CPPC(X)$_5$CDSFTNCWELLTCPPC) in consensus with the Library-3 targeting MDM2.

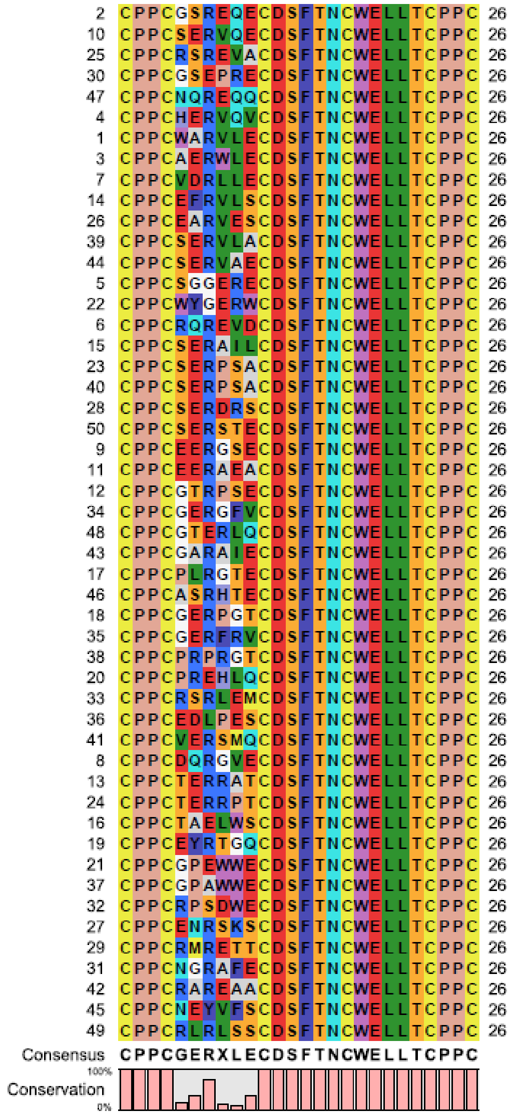

Figure S12. Selected top 50 peptide sequences (CPPC(X)$_6$CDSFTNCWELLTCPPC) in consensus with the Library-3 targeting MDM2.

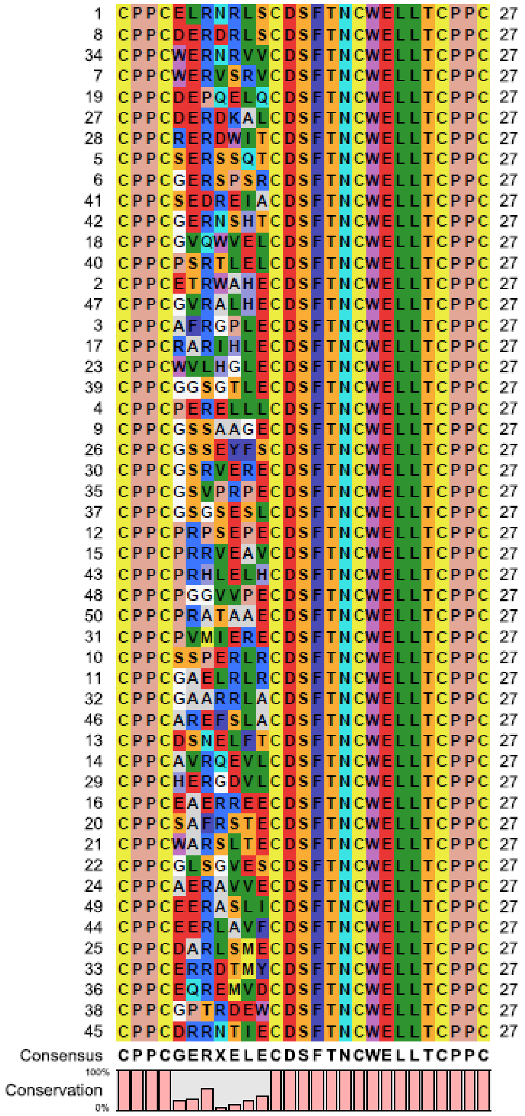

Figure S13. Selected top 50 peptide sequences (CPPC(X)$_7$CDSFTNCWELLTCPPC) in consensus with the Library-3 targeting MDM2.

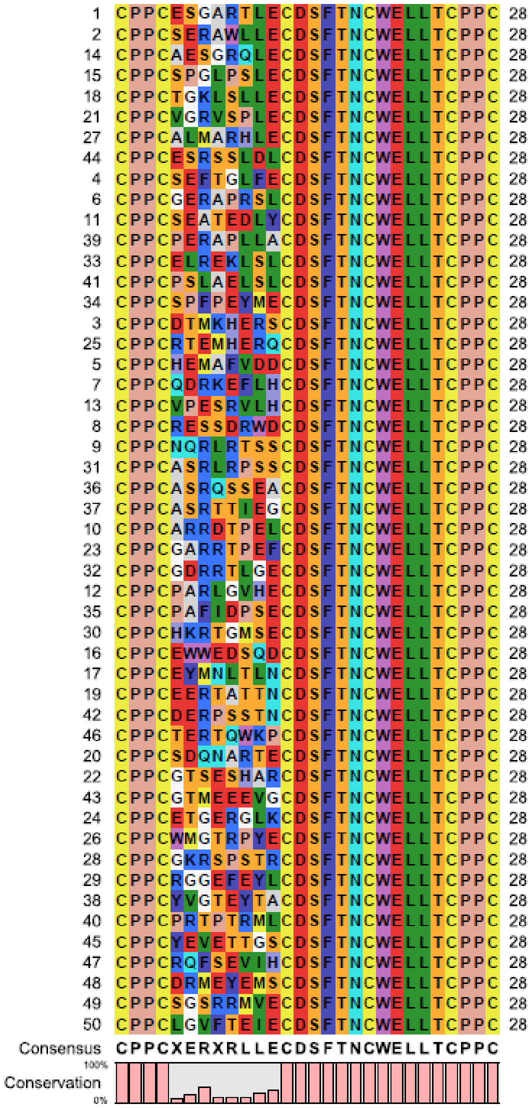

Figure S14. Selected top 50 peptide sequences (CPPC(X)$_8$CDSFTNCWELLTCPPC) in consensus with the Library-3 targeting MDM2.

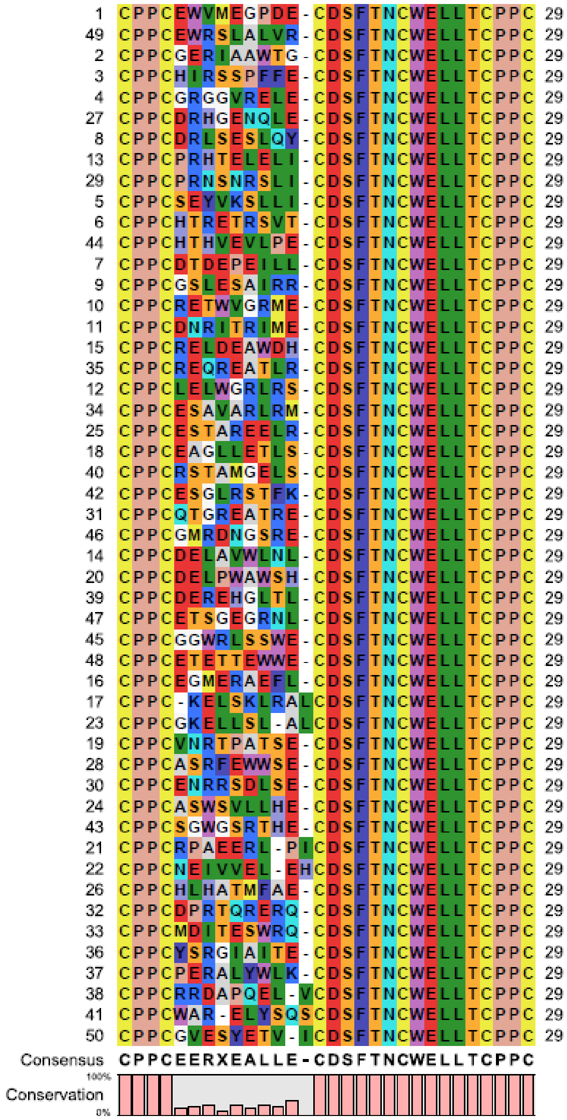

Figure S15. Selected top 50 peptide sequences (CPPC(X)₉CDSFTNCWELLTCPPC) in consensus with the Library-3 targeting MDM2.

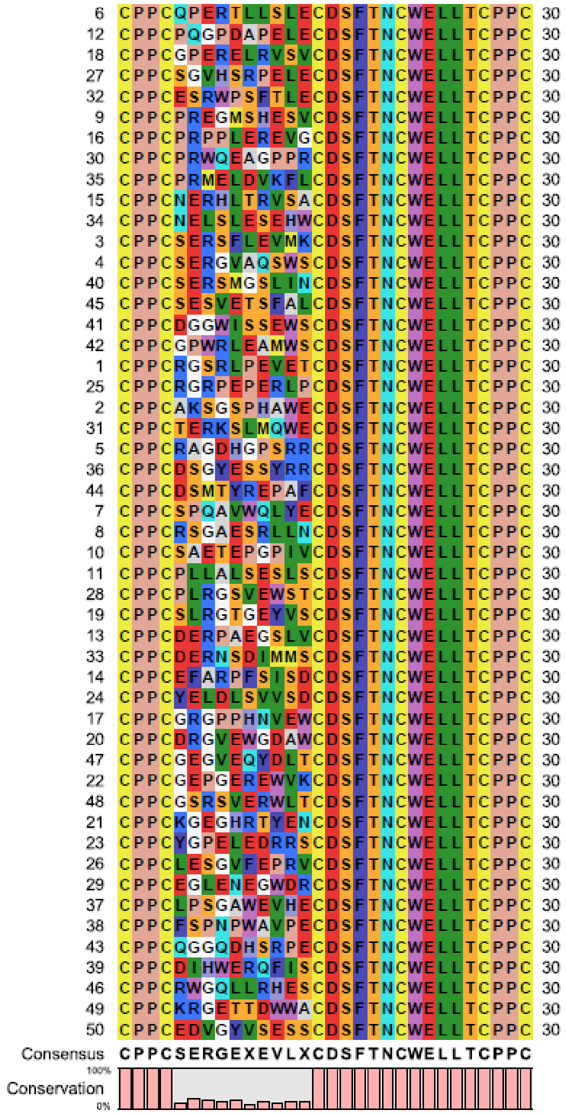

Figure S16. Selected top 50 peptide sequences (CPPC(X)$_{10}$CDSFTNCWELLTCPPC) in consensus with the Library-3 targeting MDM2.

Top 50 peptide sequences in consensus with the Library-3 targeting CD28

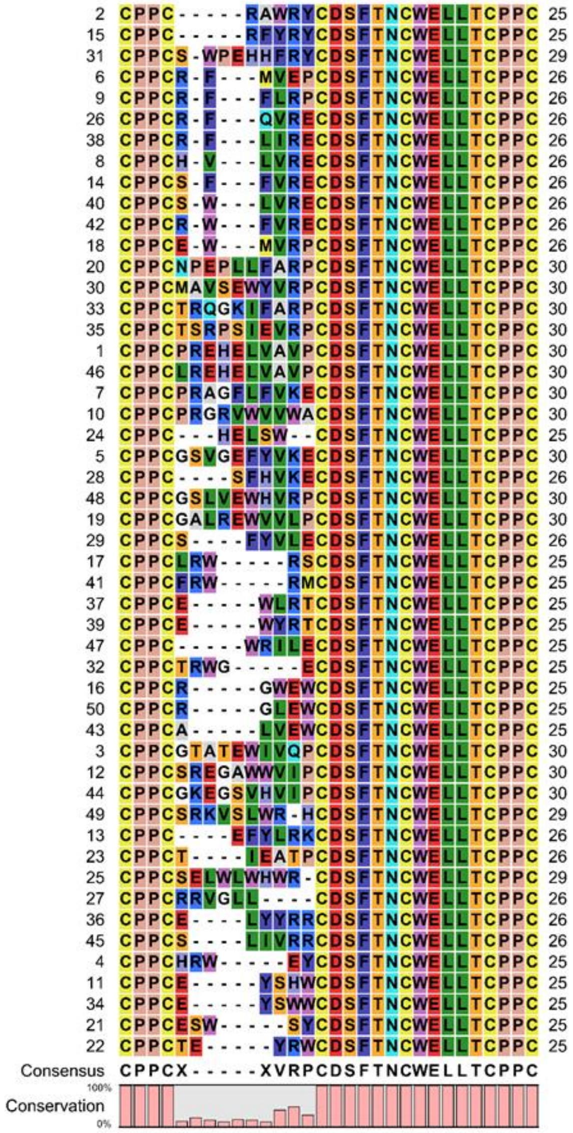

Figure S17. Selected top 50 peptide sequences in consensus with the Library-3 targeting CD28.

Screening of secondary library (Library-4) against CD28

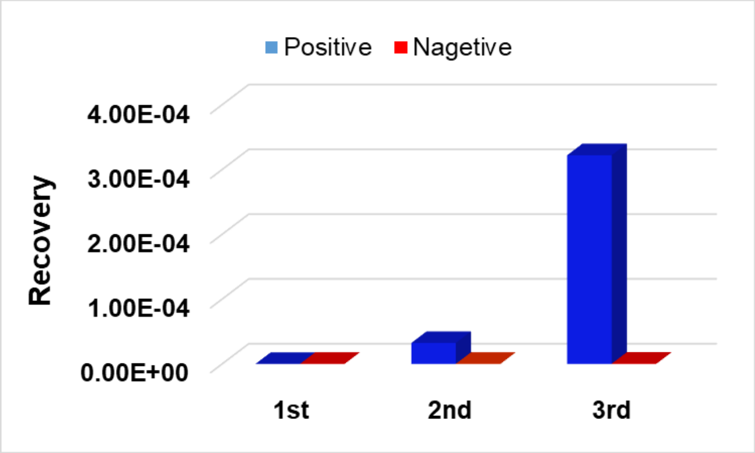

Figure S18. Screening of secondary library against CD28. (Blue bars: phage eluted from CD28 immobilized beads; red bars: phage eluted from beads without CD28). The recovery was defined as the ratio of recovered phage amount from CD28 immobilized beads (blue) or magnetic beads-only (red) to the amount of the input library. 1st: first round screening; 2nd: second round screening; 3rd: third round screening.

Top 50 peptide sequences in consensus with the Library-4 targeting CD28

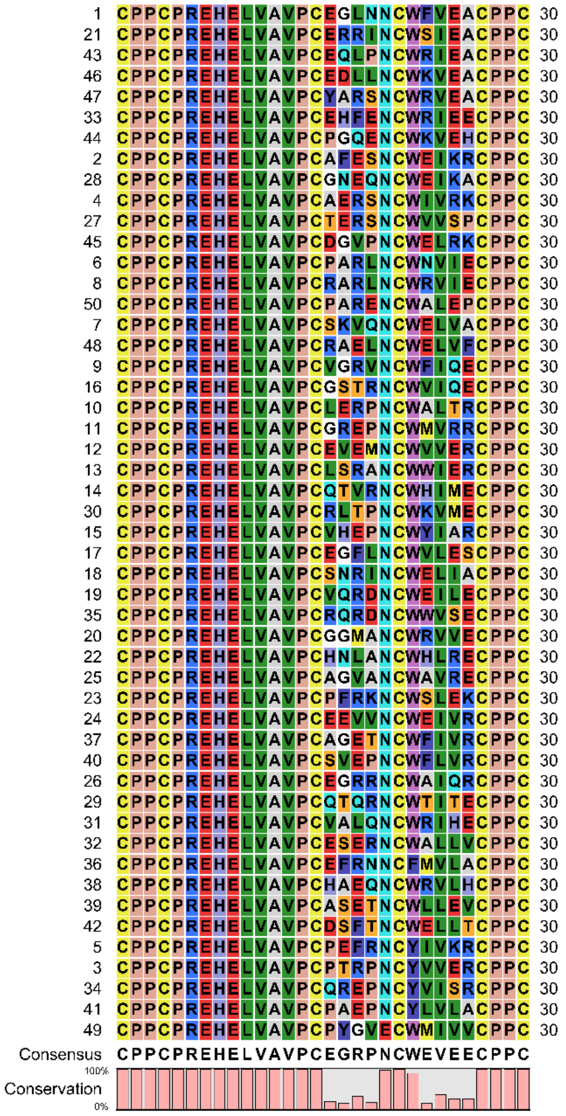

Figure S19. Selected top 50 peptide sequences in consensus with the Library-4 targeting CD28. Alignments of sequences reveal a conserved motif of NCWXV/L, with other residues flanking this motif highly diverse.

Characterization of drp7, drp8, drp9 and FITC-drp8

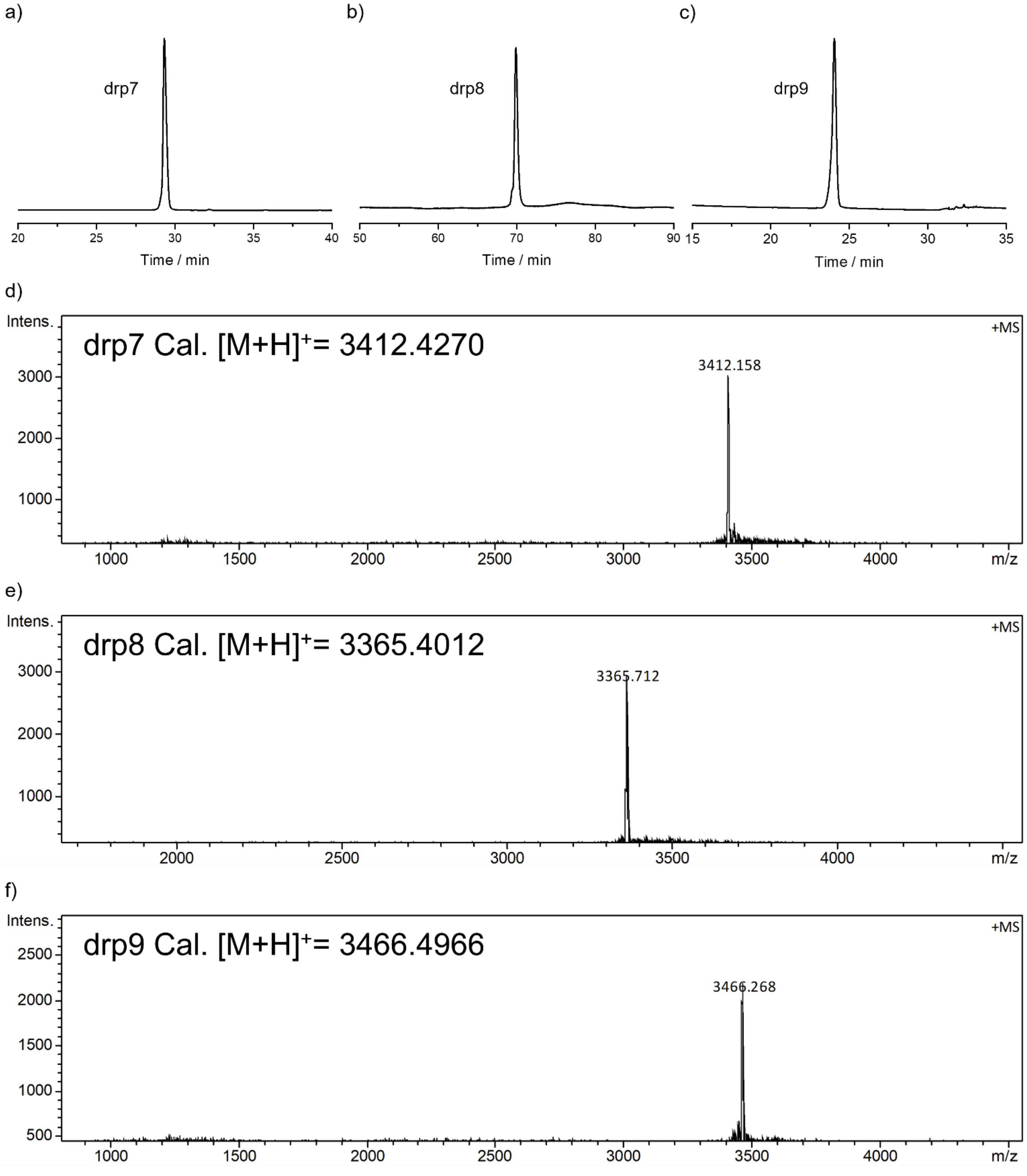

Figure S20. a) **drp7** used for FP assays was purified using HPLC to a purity of >95%. b) **drp8** used for FP assays was purified using HPLC to a purity of >95%. c) **drp9** used for FP assays was purified using HPLC to a purity of >95%. d) Mass spectrum of reduced **drp7**. e) Mass spectrum of oxidized **drp8**. f) Mass spectrum of oxidized **drp9**.

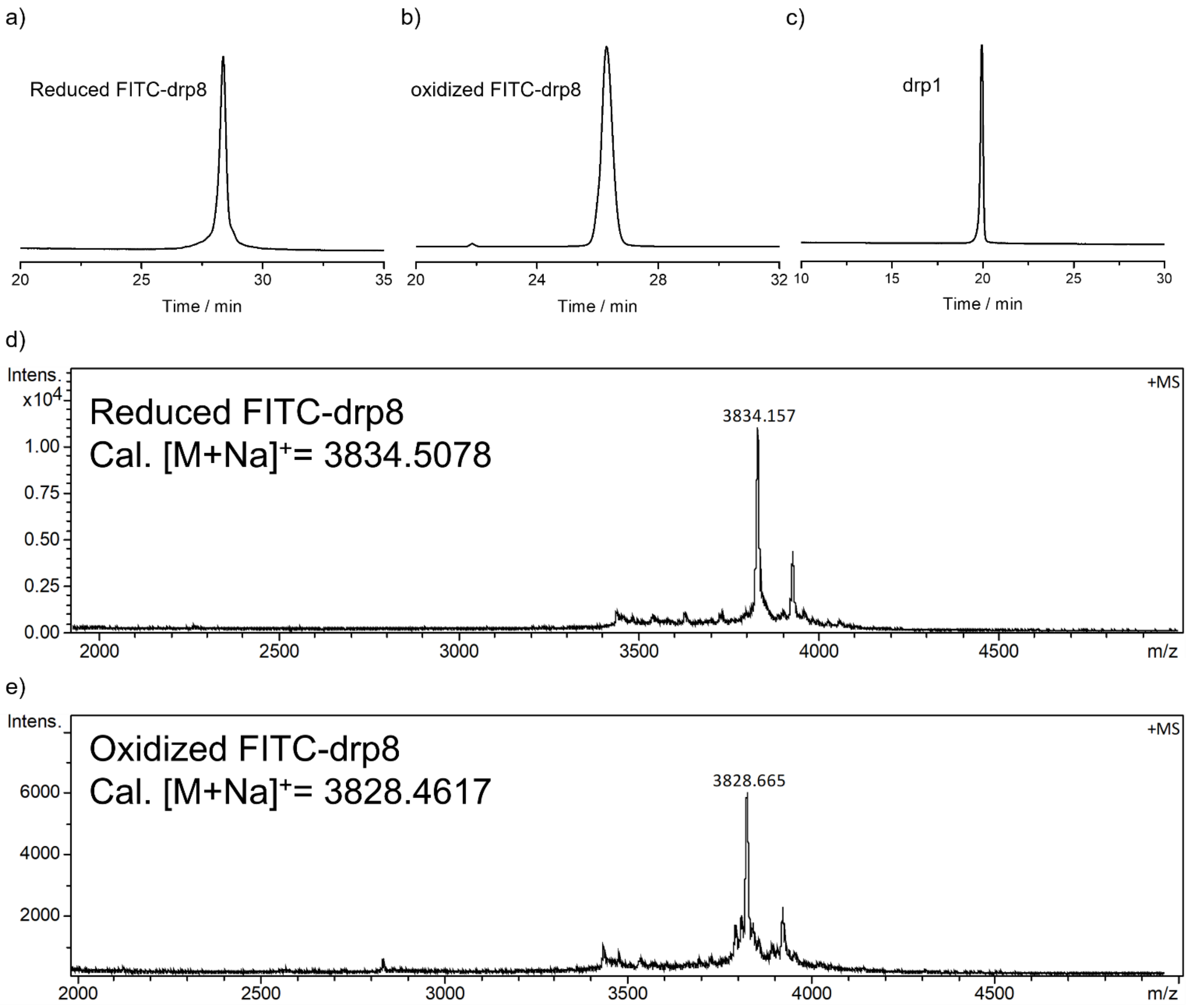

Figure S21. a) The reduced FITC-**drp8** used for FP assays was purified using HPLC to a purity of >95%. b) The oxidized FITC-**drp8** used for FP assays was purified using HPLC to a purity of >95%. c) The oxidation product **drp1** used for FP assays was purified using HPLC to a purity of >95%. d) Mass spectrum of reduced FITC-**drp8**. e) Mass spectrum of oxidized FITC-**drp8**.

Binding of CD80 to CD28

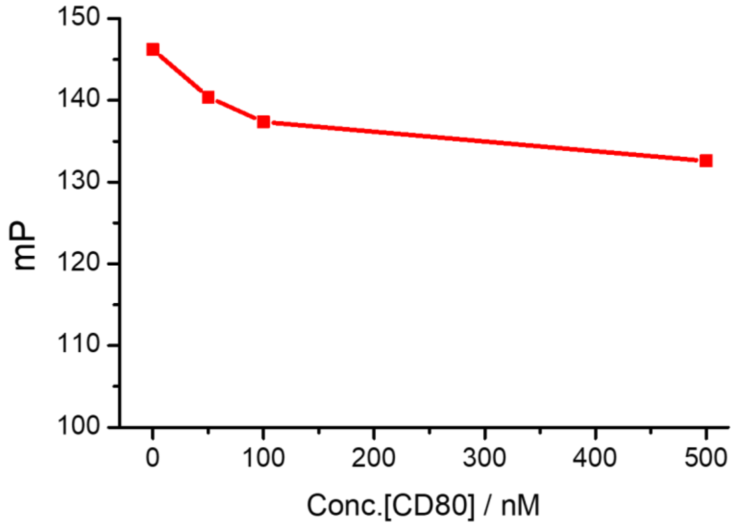

Figure S22. Binding of CD80 to CD28 determined by competing with oxidized FITC-**drp8** in a FP assay.

Binding of drp8 to CTLA-4

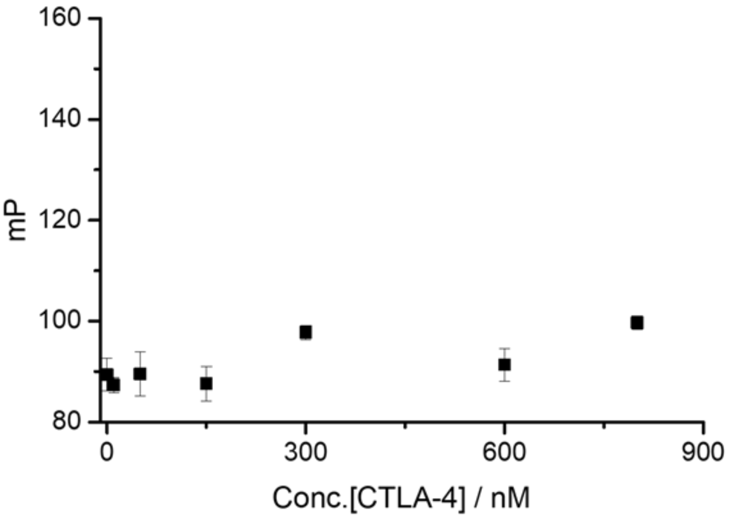

Figure S23. Binding of oxidized **drp8** labeled with fluorescein (FITC) to CTLA-4 determined using a fluorescence polarization (FP) assay.

Experimental NMR data

Table S3 NMR and refinement statistics for the structures of peptides

	drp1	drp2-a	drp2-b	drp3	drp4	drp6	drp8-I	drp8-II
**NMR distance and dihedral constraints**								
Distance constraints								
Total NOE	236	206	217	182	347	222	250	195
Intra-residue	128	129	120	94	185	113	131	111
Inter-resudue	108	77	97	88	162	109	119	84
Sequential ($\|i - j\| = 1$)	74	64	76	70	124	75	65	71
Medium-range ($\|i - j\| \leq 4$)	29	6	17	14	34	11	22	5
Long-range ($\|i - j\| \geq 5$)	5	7	4	4	4	23	32	8
Hydrogen bonds	4	0	4	0	6	0	0	0
Total dihedral angle restraints	32	30	32	28	52	46	36	44
Φ	16	15	16	14	26	23	18	22
Ψ	16	15	16	14	26	23	18	22
**Structures statistics**								
Mean restraint violations								
Distance restraint violations (>0.3 Å)	0	0.27±0.44	0.60±0.49	0.13±0.49	0	0	0.06±0.25	0.13±0.34
Dihedral restraint violations (>5°)	0	0	0	0	0.40±0.23	0	0	0
Average r.m.s. deviation* (Å)								
Backbone RMSD	0.57±0.16	2.38±0.30	1.02±0.32	2.41±0.96	1.16±0.33	1.12±0.35	1.23±0.39	1.57±0.75
Heavy atoms RMSD	1.12±0.13	2.73±0.27	1.18±0.32	2.60±0.85	1.53±0.35	1.40±0.32	1.63±0.46	2.03±0.84
**PDB code**	7W8K	7W8O	7W8R	7W8T	7W8Z	7W96	7WEI	7WE3

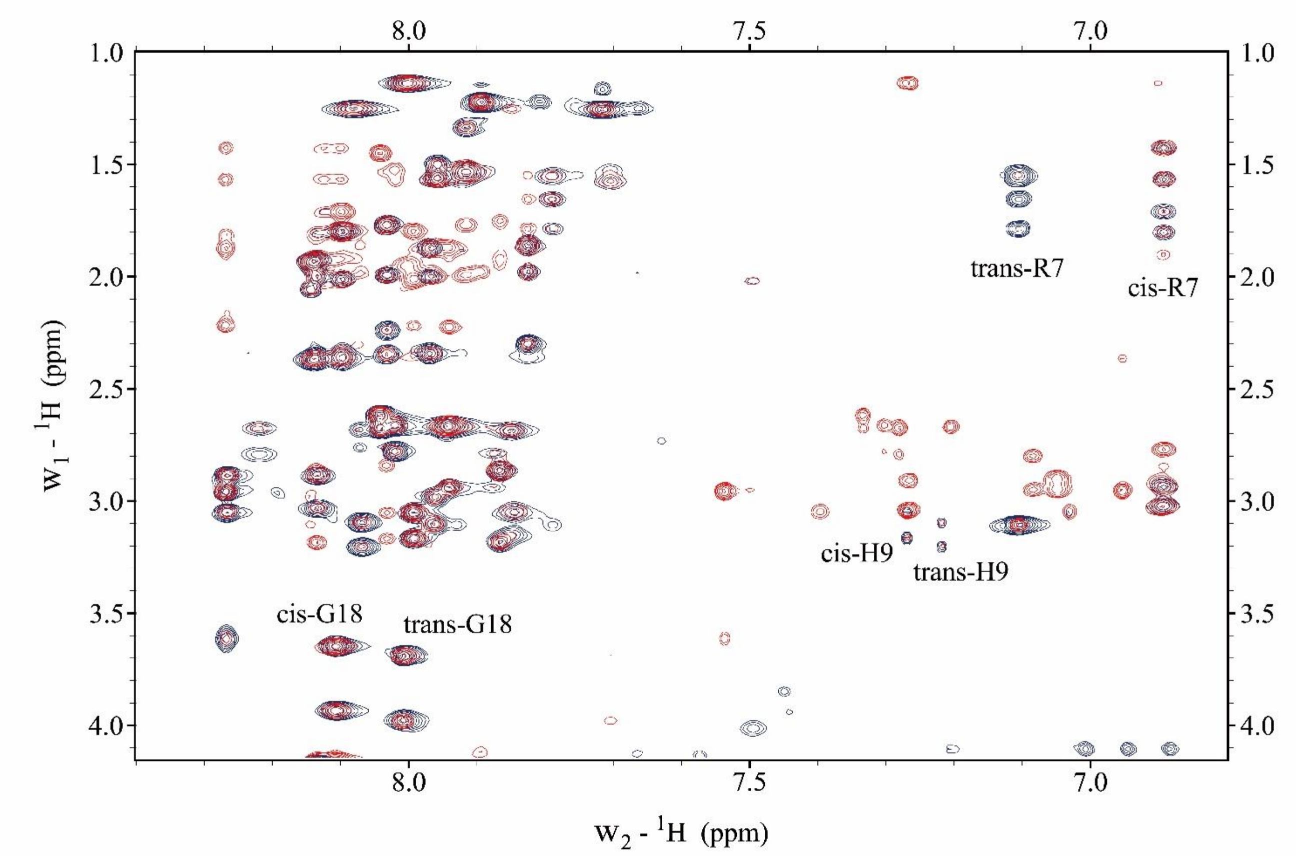

Figure S24. Two-dimensional ^{1}H-^{1}H TOCSY and ^{1}H-^{1}H NOESY spectra of **drp8**. In the right of local regions (dark blue: TOCSY, red: NOESY), Gly18, His9 and Arg7 clearly shows significantly different chemical shifts.

References

1. S. M. Lu, Y. P. Wu, J. J. Li, X. T. Meng, C. L. Hu, Y. B. Zhao and C. L. Wu, *J. Am. Chem. Soc.*, 2020, **142**, 16285-16291.
2. A. Ernst, D. Gfeller, Z. Y. Kan, S. Seshagiri, P. M. Kim, G. D. Bader and S. S. Sidhu, *Mol Biosyst*, 2010, **6**, 1782-1790.
3. I. R. Rebollo, M. Sabisz, V. Baeriswyl and C. Heinis, *Nuleic. Acids Res.*, 2014, **42**.
4. Z. Nikolovska-Coleska, R. X. Wang, X. L. Fang, H. G. Pan, Y. Tomita, P. Li, P. P. Roller, K. Krajewski, N. G. Saito, J. A. Stuckey and S. M. Wang, *Anal. Biochem.*, 2004, **332**, 261-273.
5. F. Delaglio, S. Grzesiek, G. W. Vuister, G. Zhu, J. Pfeifer and A. Bax, *J. Biomol. NMR*, 1995, **6**, 277-293.
6. Y. Shen and A. Bax, *J. Biomol. NMR*, 2013, **56**, 227-241.
7. W. Rieping, M. Habeck, B. Bardiaux, A. Bernard, T. E. Malliavin and M. Nilges, *Bioinformatics*, 2007, **23**, 381-382.
8. A. T. Brunger, P. D. Adams, G. M. Clore, W. L. DeLano, P. Gros, R. W. Grosse-Kunstleve, J. S. Jiang, J. Kuszewski, M. Nilges, N. S. Pannu, R. J. Read, L. M. Rice, T. Simonson and G. L. Warren, *Acta Crystallogr. D Biol. Crystallogr.*, 1998, **54**, 905-921.
9. R. A. Laskowski, J. A. C. Rullmann, M. W. MacArthur, R. Kaptein and J. M. Thornton, *J. Biomol. NMR*, 1996, **8**, 477-486.
